# Supplementary material for: Learning a chemistry-aware latent space for molecular encoding and generation with a large-scale transformer variational autoencoder
Source: J Cheminform. 2026 Jun 16;18:123. doi: 10.1186/s13321-026-01243-0 (PMC13508326; doi:10.1186/s13321-026-01243-0)
Supplement: Supplementary file 1 [file 13321_2026_1243_MOESM1_ESM.pdf]

Additional file 1: Learning a chemistry-aware latent space for molecular encoding and generation with a large-scale Transformer Variational Autoencoder

Hugo Talibart, Dimitri Gilis
Contact: hugo.talibart@ulb.be

1 Implementation details

Our implementation is based on PyTorch and PyTorch Lightning using Python 3.12. The maximum SELFIES length was set to 256. The encoder includes 6 Transformer encoder layers with multi-head self-attention (8 heads- and feed-forward networks (hidden dimension 1024) with ReLU activation, layer normalization and dropout (0.1). The latent embedding has dimensionality 256. The decoder includes 6 Transformer decoder layers with masked multi-head self-attention (8 heads), cross-attention and feed-forward networks (hidden dimension 512) with ReLU activation, layer normalization and dropout. In total, the autoencoder has 17.6 M Parameters. The regressor is a multilayer perceptron with two hidden layers (dimensions resp. 32 and 16) with ReLU activations, for a total of 8.9 K parameters.

The base model was trained with the Adam optimizer using a batch size of 256, a learning rate of 10^{-4} and a warm-up schedule of 100 steps. To improve training stability, gradient values were clipped to 1.0 to avoid exploding gradients, and the decoder and the regressor were trained only every 5 steps. Weight decay was applied to all layers except for the embedding layers with a rate of 10^{-5}. Pre-training did not use a fixed number of epochs or early stopping: we monitored the validation loss and stopped when it reached a plateau, which occurred after approximately 3.41 M steps (22 epochs). Training was performed on a single NVIDIA RTX 5000 Ada GPU, using medium float32 precision to improve efficiency. For fine-tuning, models were trained with a learning rate of 10^{-5} for a fixed 200 epochs for simplicity.

2 SELFIES token semantic distance

To initialize embedding layers and handle the encoding of tokens that are not in the initial vocabulary, we define a custom distance metric between SELFIES tokens that reflect their semantic meanings. Notice that each SELFIES tokens are follows a consistent format:

1. Opening bracket `[`

2. Bond indicator: one of `#` (triple), `=` (double), `-/`, `-\`, `/`, `\` or none (single)

3. Main symbol: an atomic element (e.g. `C`, `Br`) or a topology marker (e.g. `Branch1`, `Ring2`)

4. Chirality indicator: `@` or `@@` or none

5. Explicit hydrogen count: (`H`, `H0`, `H1`, etc. or none)

6. Charge indicator: `+1`, `-1`, `+2`, etc. or none

7. Closing bracket `]`

We define the distance between two tokens as a weighted sum of component-wise distances, each rescaled to be between 0 and 1:

- **Bond distance**: 0 if bond indicators match exactly, 0.1 if they differ only in stereochemistry but represent the same bond order, otherwise the average bond order. Weighted by 2.

- **Main symbol distance**: 0 if identical, 0.9 if both atoms or topological tokens, 1 otherwise. Weighted by 5.

- **Chirality distance**: 0 if chirality indicators match, 1 otherwise. Weighted by 1.

- **Hydrogen distance**: 1 if one token has no explicit hydrogen and the other one does, otherwise, the absolute difference in hydrogen count divided by 4. Weighted by 1.

- **Charge distance**: Absolute charge difference divided by 5. Weighted by 1.

3 Ablation study

3.1 Dataset construction

Because the original training set was too large for practical ablation studies, we derived a representative subset using stratified sampling. Molecules were stratified by SELFIES length ("short": $< \frac{1}{3}$ quantile = 29, "long": $> \frac{2}{3}$ quantile = 45, "medium": in between) and by presence/absence of rare tokens (defined as occurring $< 100\,000$ times in the training set), yielding 6 strata. A balanced sample of 1 000 000 molecules was drawn and split into training (80%) and validation (20%) sets. Oversampling was performed following the strategy described in section 2.5 of the main text.

3.2 Experimental setup

We used the same architecture and hyperparameters as the main model, differing only in the training objective. Starting from a baseline with reconstruction loss and KL coefficient of 0.2, we varied the Tanimoto loss coefficient $\beta_{Tanimoto}$ (0, 0.2, 0.4, 0.6, 0.8, 1.0), the KL coefficient β_{KL} (0, 0.2, 0.4, 0.6, 0.8, 1.0, 2.0), and the property loss coefficient β_{prop} (0, 0.2, 0.4, 0.6, 0.8, 1.0). All experiments were ran for 5 epochs with 3 different random seeds.

3.3 Evaluation metrics

After training, each model was evaluated on reconstruction, generation, and latent space structure: reconstruction on the full validation set, generation on 10 000 sampled molecules, and latent space structure on 2 000 validation molecules randomly sampled. Specifically, we measured:

- Token reconstruction accuracy
- KL divergence loss
- Uniqueness
- Internal diversity ($IntDiv_1$, $IntDiv_2$)
- SELFIES, molecule and fingerprint novelty scores
- Pearson correlation between Euclidean distances in latent space and Tanimoto similarities
- Pearson correlation between Euclidean distances in latent space and absolute difference of molecular properties
- Trustworthiness, a local measure of neighborhood preservation defined in [2] as:

$$T(k) = 1 - \frac{2}{nk\left(2n - 3k - 1\right)} \sum_{i=1}^{n} \sum_{j \in \mathcal{N}_i^k} \max\left(0, \left(r_i\left(j\right) - k\right)\right) \tag{S1}$$

  where n is the number of sampled molecules, $\mathcal{N}_i^k$ is the set of the k nearest neighbors of molecule i in latent space in terms of Euclidean distance, and $r_i(j)$ is the rank of molecule j in the chemical space neighborhood of i, measured by Tanimoto similarity. Intuitively, whenever a latent space neighbor j is not among the k closest molecules to i in chemical space, it is penalized in proportion to its rank. We used $k = 5$, $k = 10$, $k = 20$ and $k = 30$ neighbors.

3.4 Additional results

As shown in figure S1, the KL divergence coefficient critically determines reconstruction quality. As it increases, token reconstruction accuracy decreases. For large coefficients, a posterior collapse occurs when the objective function contains only reconstruction and KL losses: the decoder outputs overly generic outputs that match tokens well on average but miss the finer structural distinctions between molecules, leading to a higher token reconstruction accuracy but a significant drop in the uniqueness of generated molecules. Including the property regression loss prevents this collapse, showing its role as regularizer.

Furthermore, although the Tanimoto loss is the main driver of latent space organization, ablations without it – varying only the properties loss coefficient β_{prop} (figure S2) – show that increasing β_{prop} draws molecules with similar properties closer together in latent space, with

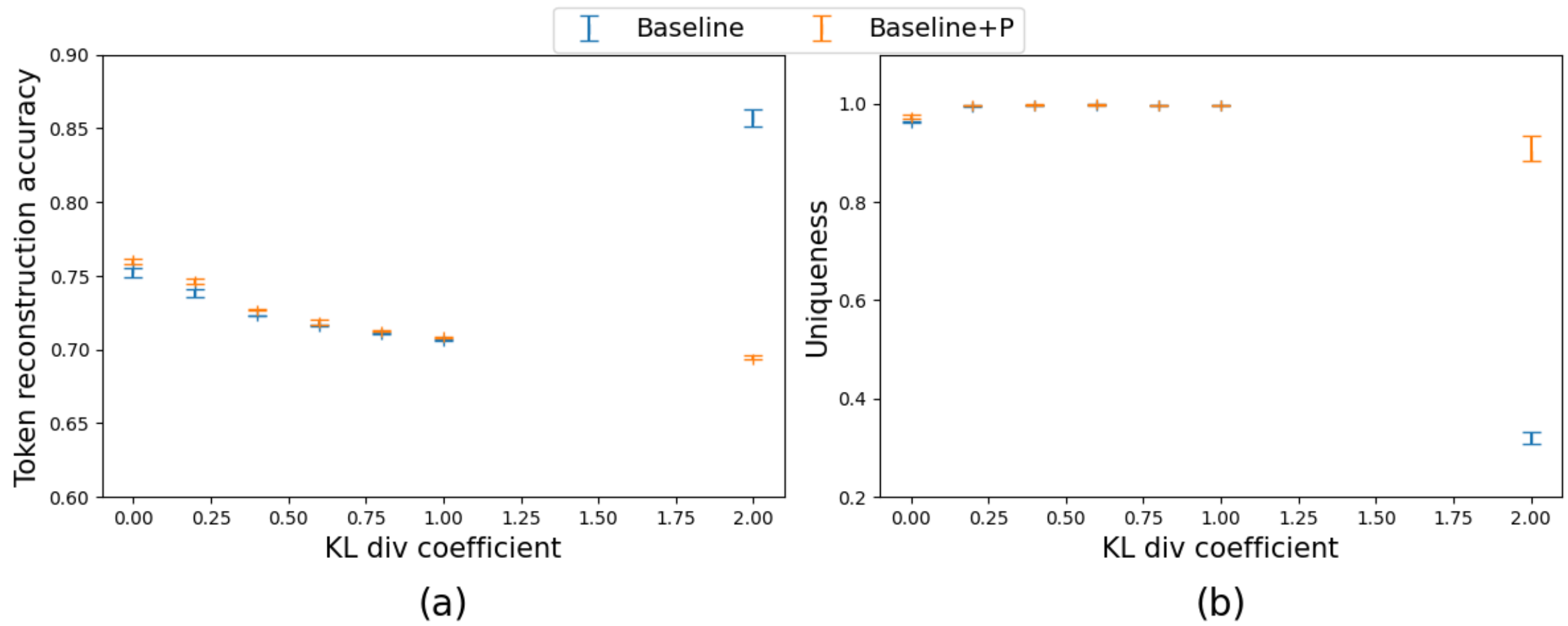

Figure S1: **Impact of the KL divergence loss coefficient.** (a) Token reconstruction accuracy versus KL divergence coefficient (b) Uniqueness of generated molecules versus KL divergence coefficient. Results are shown for the baseline (only reconstruction loss and KL divergence loss) in blue and for the baseline with the properties loss in orange. Each point shows the median across 3 seeds, with error bars indicating the range.

a modest secondary effect on the correlation between Tanimoto similarities and Euclidean distances, whose absolute value increases from 0.39 to 0.43.

Aside from the case of posterior collapse at high KL coefficients, generation diversity (uniqueness, IntDiv1, IntDiv2) and novelty (SELFIES, molecule, fingerprint) scores remained stable across experiments, showing no clear trend, as shown in figure S3.

4 Molecular optimization additional results

For each task, we report the generated molecules with their fitness, SA score, and Tanimoto similarity to the initial molecules, along with the best molecule(s) in the initial dataset where applicable, pairwise Tanimoto and scaffold Tanimoto similarities (Tanimoto similarities between Bemis-Murcko scaffolds [1]) between initial and generated molecules where applicable, fitness distributions, and SA score and QED distributions.

4.1 Design of Organic Photovoltaics

Results for the $PCE_{PCBM} - SA_{score}$ and $PCE_{PCDTBT} - SA_{score}$ tasks are shown in figures S4–S11, fitness distributions in figure S12, SA score and QED distributions in figure S13, and PCE vs. SA score scatter plots in figure S14.

4.2 Design of Organic Emitters

Results for the singlet-triplet and oscillator strength tasks are shown in figures S15–S20, fitness distributions in figure S21, and SA score and QED distributions in figure S22. For

the oscillator strength task, the model outputted the starting molecule (figure S19) in 2 out of the 5 runs, and the molecule in figure S20 in the 3 other runs, which has a Tanimoto similarity and a scaffold similarity of resp. 0.33 and 0.00 with the initial molecule.

4.3 Design of Protein Ligands

Results for the 1SYH, 6Y2F and 4LDE tasks are shown in figures S23–S31, fitness distributions in figure S32, and SA score and QED distributions in figure S33. For 4LDE, the initial dataset contains 1056 molecules with best affinity (-12.2) and are not shown.

5 Latent dimension specialization analysis

To investigate whether different dimensions of the latent space specialize in encoding different types of information, we encoded 1 000 000 random molecule pairs (m_1, m_2) from the test set into latent embeddings (z_1, z_2) and computed, for each dimension $i \in \{1, \cdots, 256\}$, the Pearson correlation between the absolute differences $|z_1^i - z_2^i|$ along that dimension and the Tanimoto similarity $T(m_1, m_2)$ between the initial molecules. Interestingly, as shown in figure S34, some dimensions are more correlated with Tanimoto information as others: the correlation varies between 0.001 and 0.386.

To assess whether dimensions more correlated with Tanimoto similarity are also more critical for reconstruction, we sampled 10 000 additional molecules from the test set, encoded them, and zeroed out either the $k = 5$ dimensions most correlated with Tanimoto similarity or the $k = 5$ dimensions least correlated. Dropping the most correlated dimensions caused the string reconstruction accuracy to drop from 0.97 to 0.69, while dropping the least correlated dimensions caused only a modest drop to 0.93. This suggests that the dimensions most correlated with the Tanimoto information are also critical for reconstruction, arguing against the hypothesis that the latent space specializes into separate reconstruction and Tanimoto-encoding dimensions.

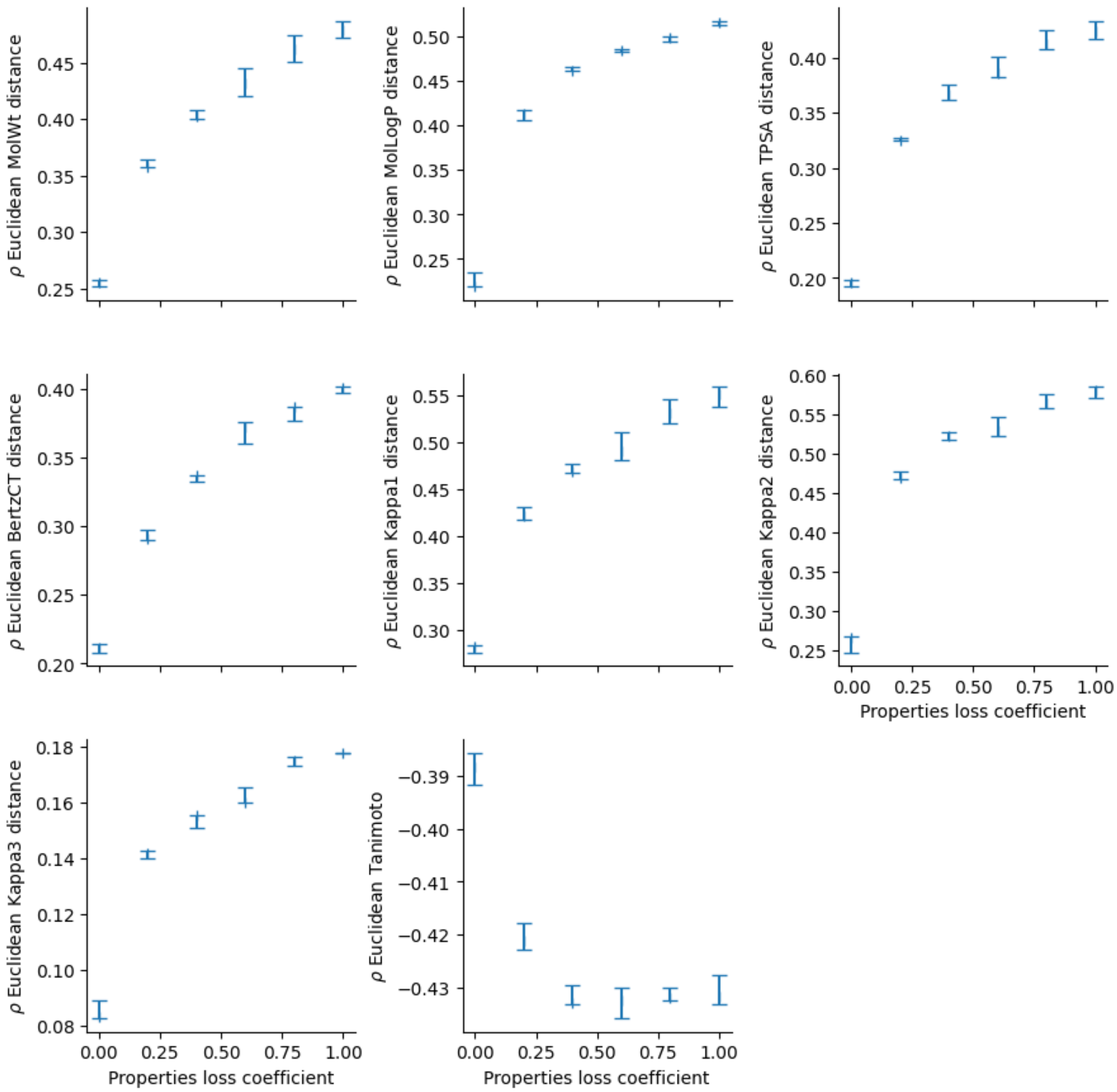

Figure S2: **Impact of the properties loss on latent space structure.** Pearson correlation between Euclidean distances in latent space and absolute value of differences in molecular property values in chemical space for all regressed properties (molecular weight, logP coefficient, polar surface area, Bertz CT and Kappa 1, Kappa 2, Kappa 3 indexes) and Tanimoto similarity. Results are shown for the baseline without Tanimoto loss, with KL divergence coefficient $\beta_{KL} = 0.2$, for properties loss coefficients β_{prop} between 0 and 1.

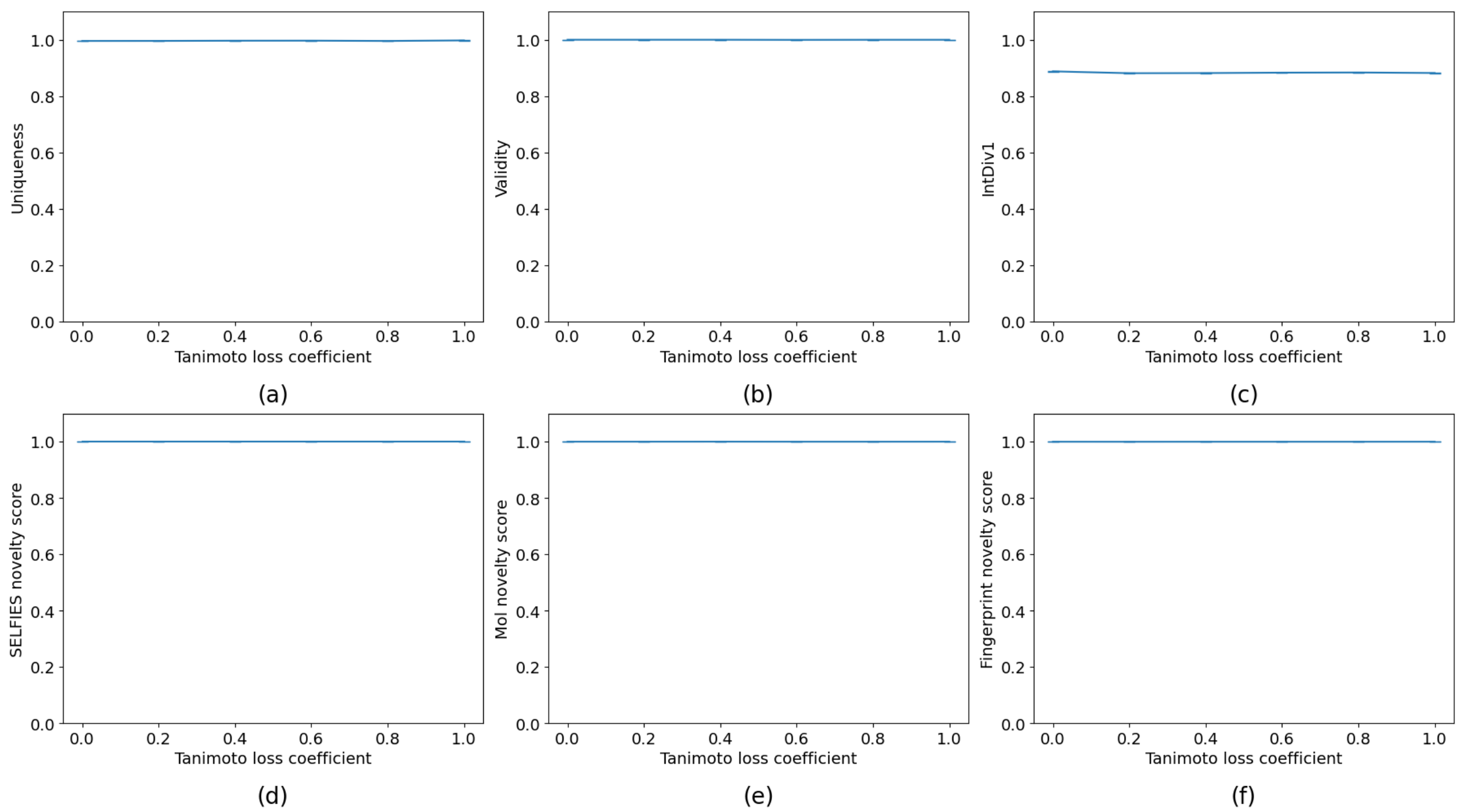

Figure S3: **Impact of Tanimoto loss coefficient on generation quality.** (a) Uniqueness, (b) validity, (c) internal diversity (IntDiv1), (d) SELFIES novelty, (e) molecular novelty, and (f) fingerprint novelty of generated molecules versus Tanimoto loss coefficient. Results are shown for the baseline with $\beta_{KL} = 0.2$ and no properties loss. Each curve shows the median across 3 seeds, with error bars indicating the range.

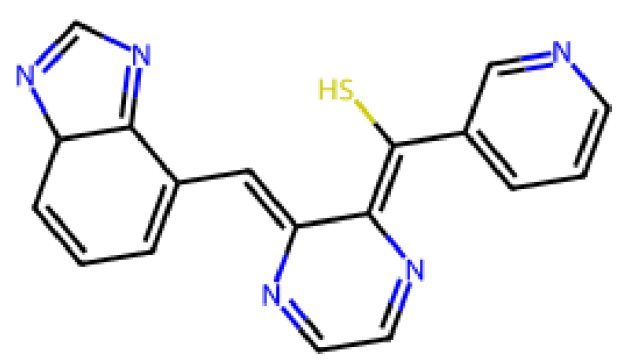

(a) Fitness: 8.40
SA_{score}: 4.59
Tanimoto: 0.13

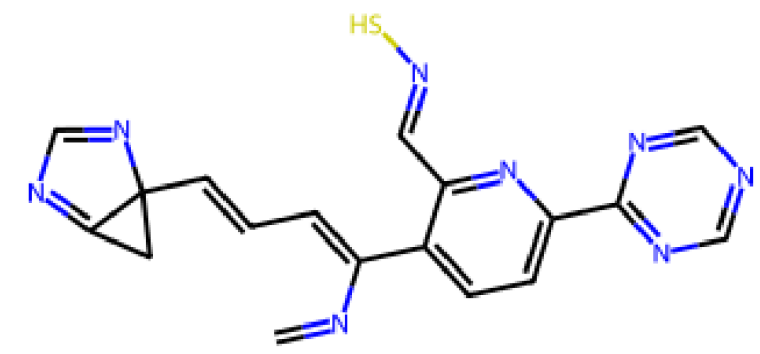

(b) Fitness: 7.58
SA_{score}: 5.14
Tanimoto: 0.19

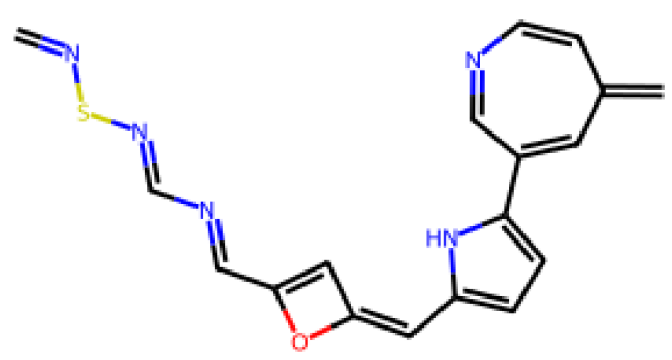

(c) Fitness: 8.27
SA_{score}: 5.08
Tanimoto: 0.10

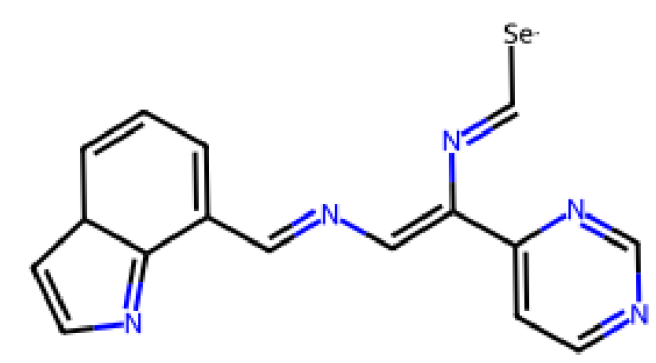

(d) Fitness: 8.91
SA_{score}: 5.09
Tanimoto: 0.11

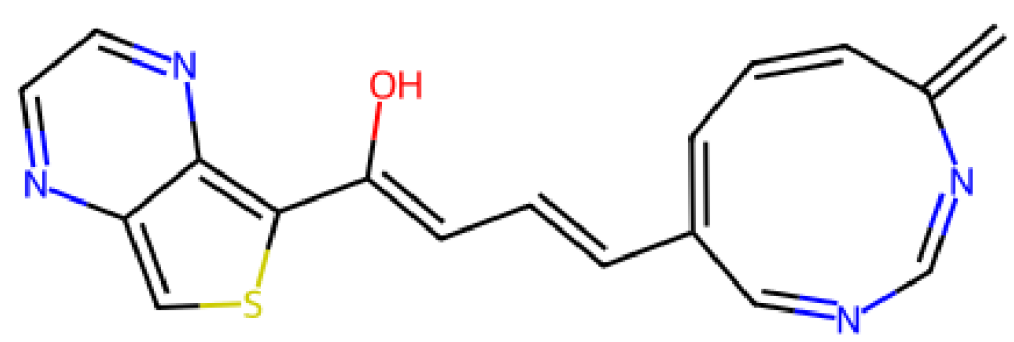

(e) Fitness: 9.14
SA_{score}: 4.68
Tanimoto: 0.14

Figure S4: **Generated molecules for the $PCE_{PCBM} - SA_{score}$ task.** Molecules generated by our model for the 5 independent runs on the $PCE_{PCBM} - SA_{score}$ maximization task.

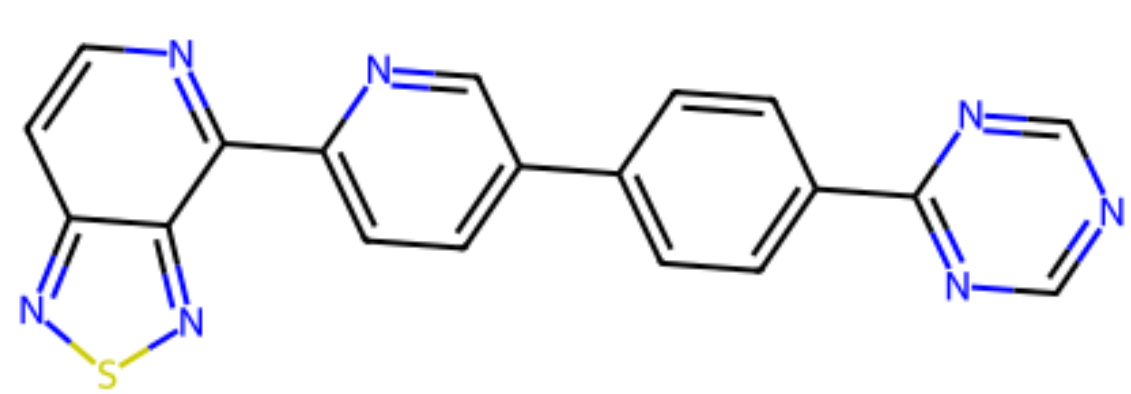

Figure S5: **Best dataset molecule for the $PCE_{PCBM} - SA_{score}$ task.** Molecule in the dataset with the highest fitness score for the $PCE_{PCBM} - SA_{score}$ maximization task, with fitness score 7.57 and SA_{score} 2.72

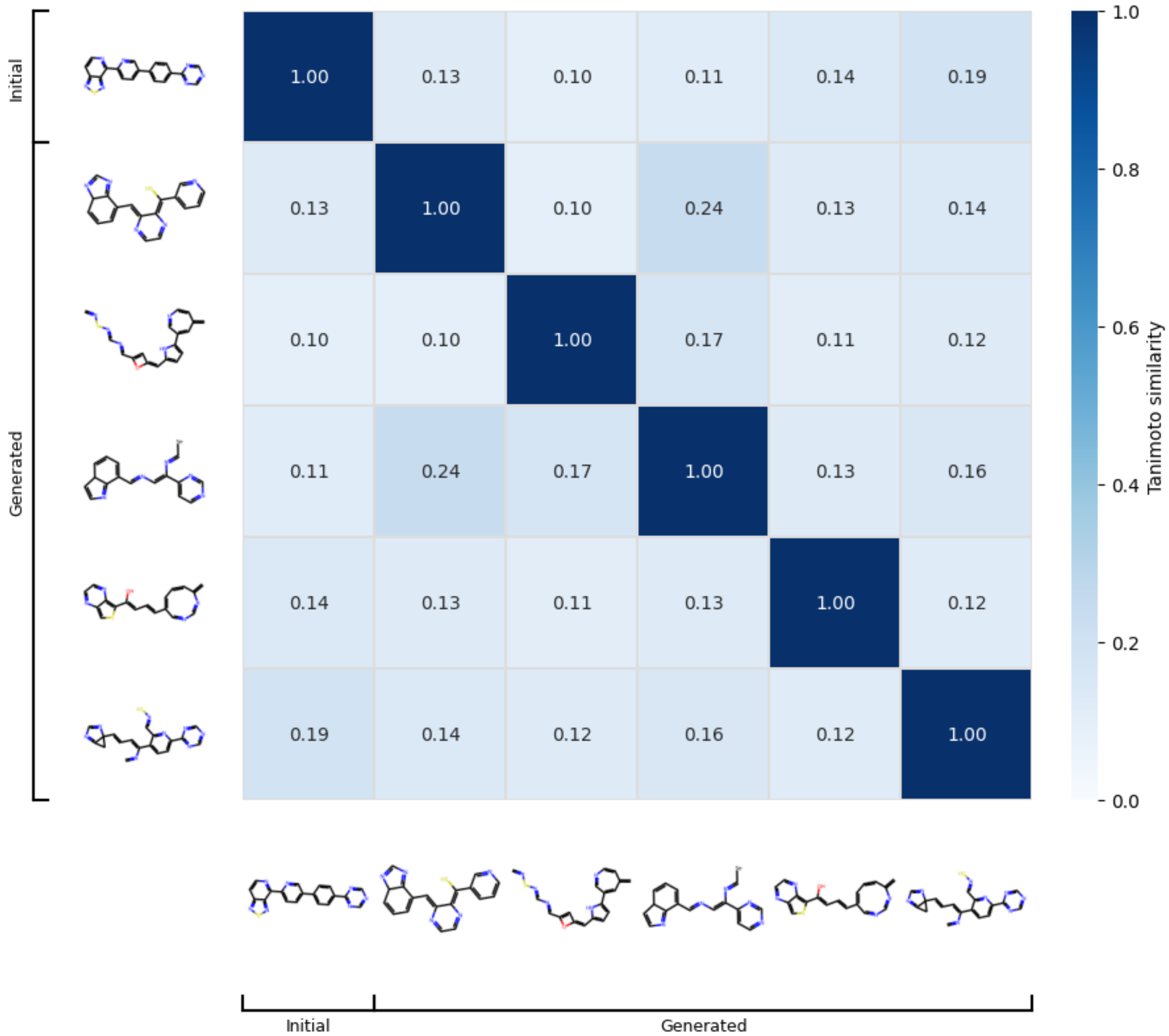

Figure S6: **Tanimoto similarity matrix for the $PCE_{PCBM} - SA_{score}$ task.** Matrix of pairwise Tanimoto similarities between initial and generated molecules for the $PCE_{PCBM} - SA_{score}$ task.

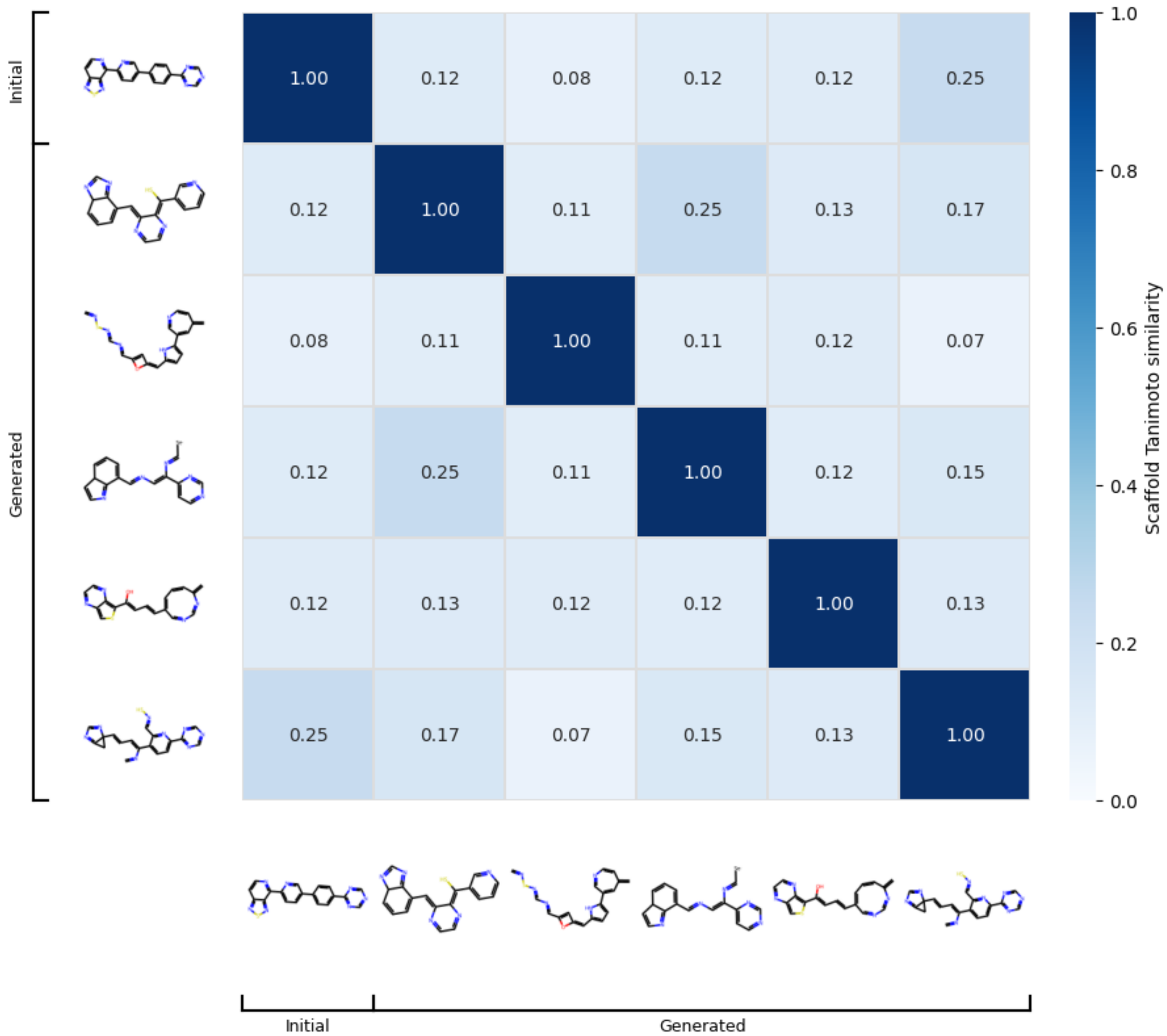

Figure S7: **Scaffold similarity matrix for the $PCE_{PCBM} - SA_{score}$ task.** Matrix of pairwise scaffold similarities between initial and generated molecules for the $PCE_{PCBM} - SA_{score}$ task.

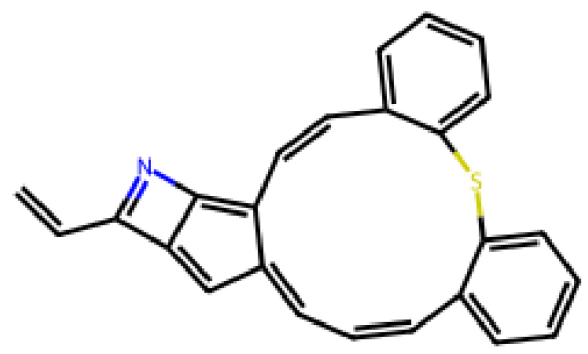

(a) Fitness: 28.29
SA_{score}: 2.90
Tanimoto: 0.18

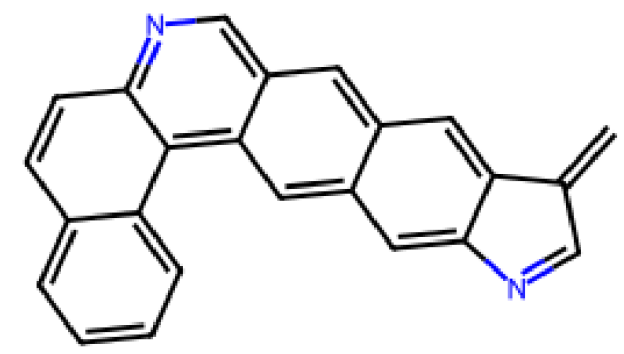

(b) Fitness: 26.95
SA_{score}: 2.90
Tanimoto: 0.53

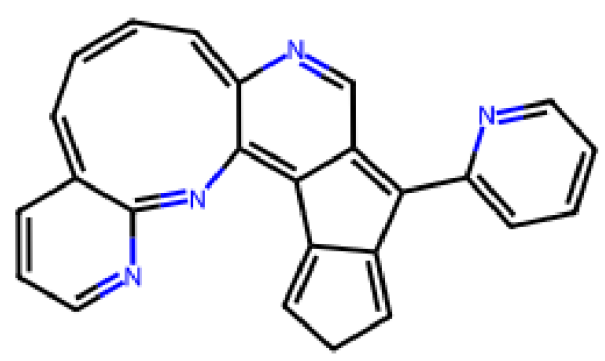

(c) Fitness: 28.65
SA_{score}: 4.35
Tanimoto: 0.17

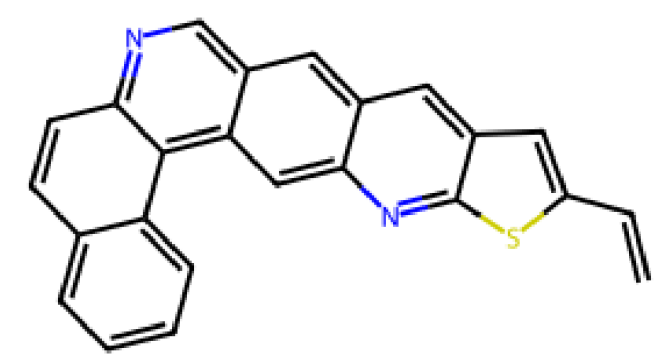

(d) Fitness: 27.09
SA_{score}: 2.85
Tanimoto: 0.55

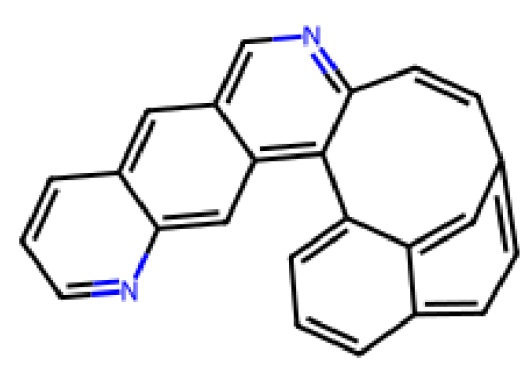

(e) Fitness: 29.22
SA_{score}: 3.52
Tanimoto: 0.47

Figure S8: **Generated molecules for the $PCE_{PCDTBT} - SA_{score}$ task.** Molecules generated by our model for the 5 independent runs on the $PCE_{PCDTBT} - SA_{score}$ maximization task.

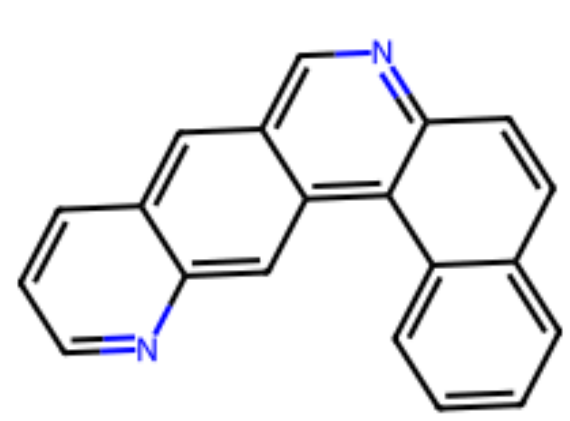

Figure S9: **Best dataset molecules for the $PCE_{PCDTBT} - SA_{score}$ task.** Molecule in the dataset with the highest fitness score for the $PCE_{PCDTBT} - SA_{score}$ maximization task, with fitness score 31.71 and SA_{score} 2.20

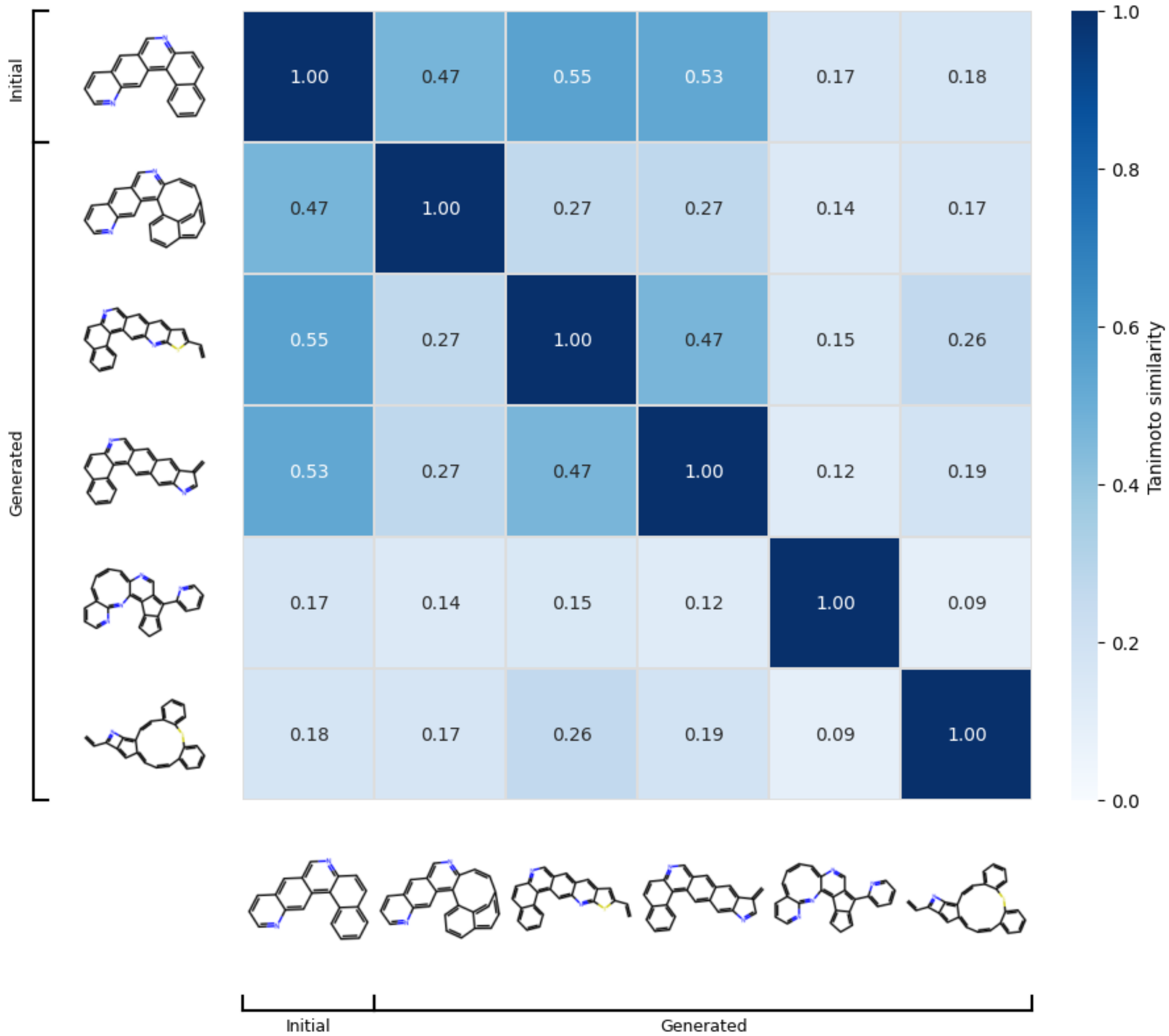

Figure S10: **Tanimoto similarity matrix for the $PCE_{PCDTBT} - SA_{score}$ task.** Matrix of pairwise Tanimoto similarities between initial and generated molecules for the $PCE_{PCDTBT} - SA_{score}$ task.

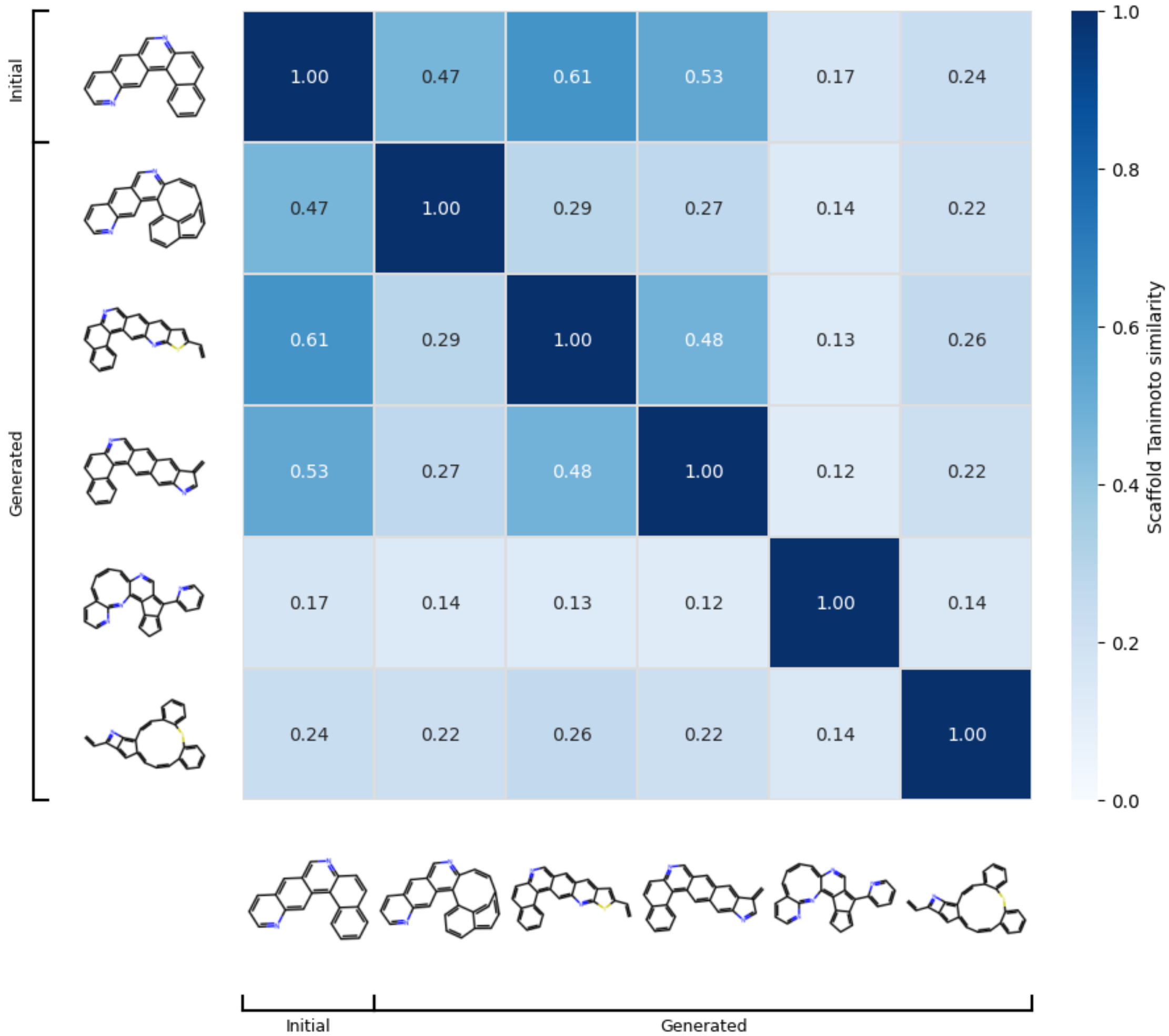

Figure S11: **Scaffold similarity matrix for the $PCE_{PCDTBT} - SA_{score}$ task.** Matrix of pairwise scaffold similarities between initial and generated molecules for the $PCE_{PCDTBT} - SA_{score}$ task.

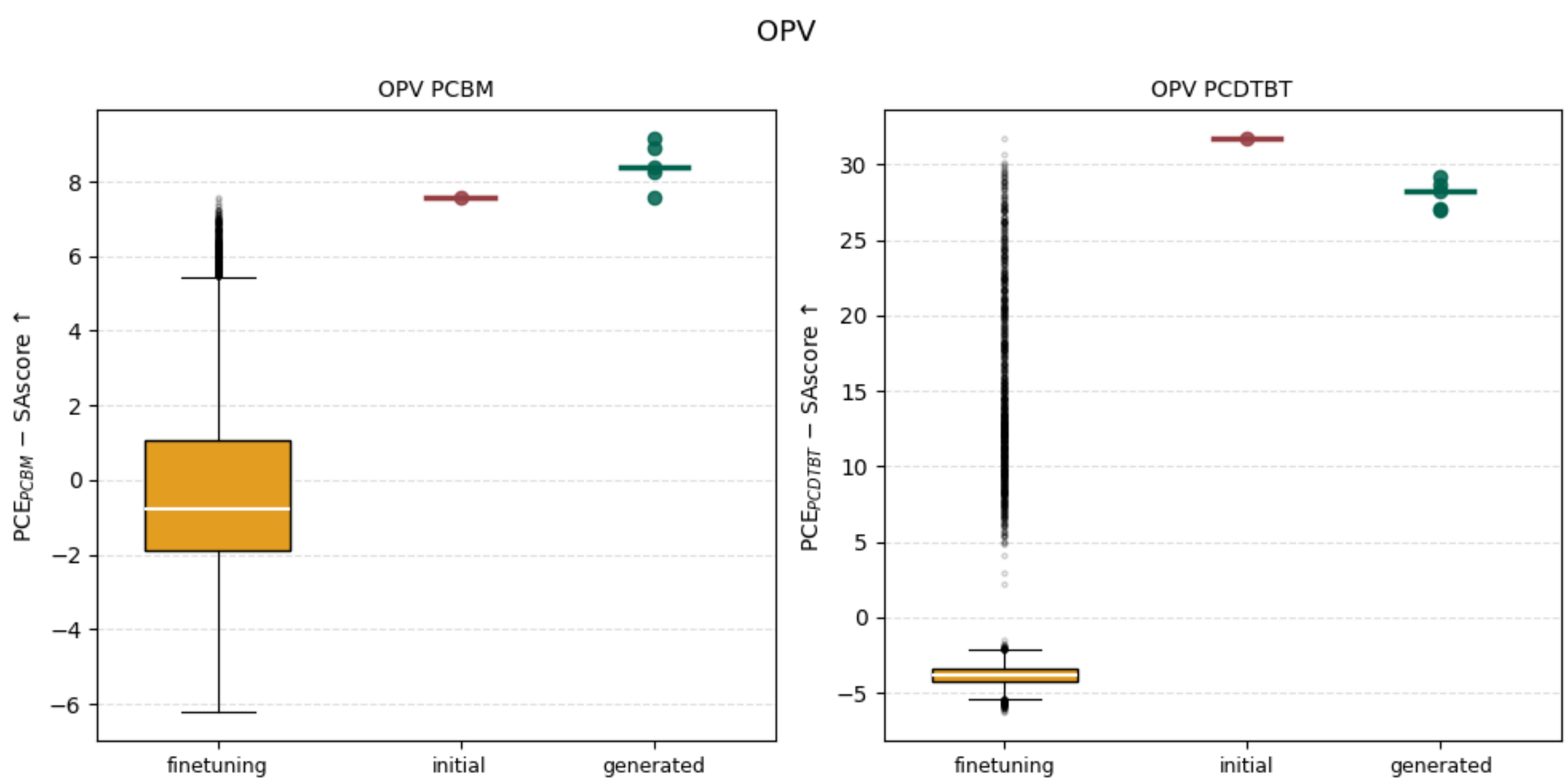

Figure S12: **Fitness distributions for the OPV tasks.** Fitness distributions for the finetuning dataset, initial and generated molecules for the $PCE_{PCBM} - SA_{score}$ and $PCE_{PCDTBT} - SA_{score}$ tasks.

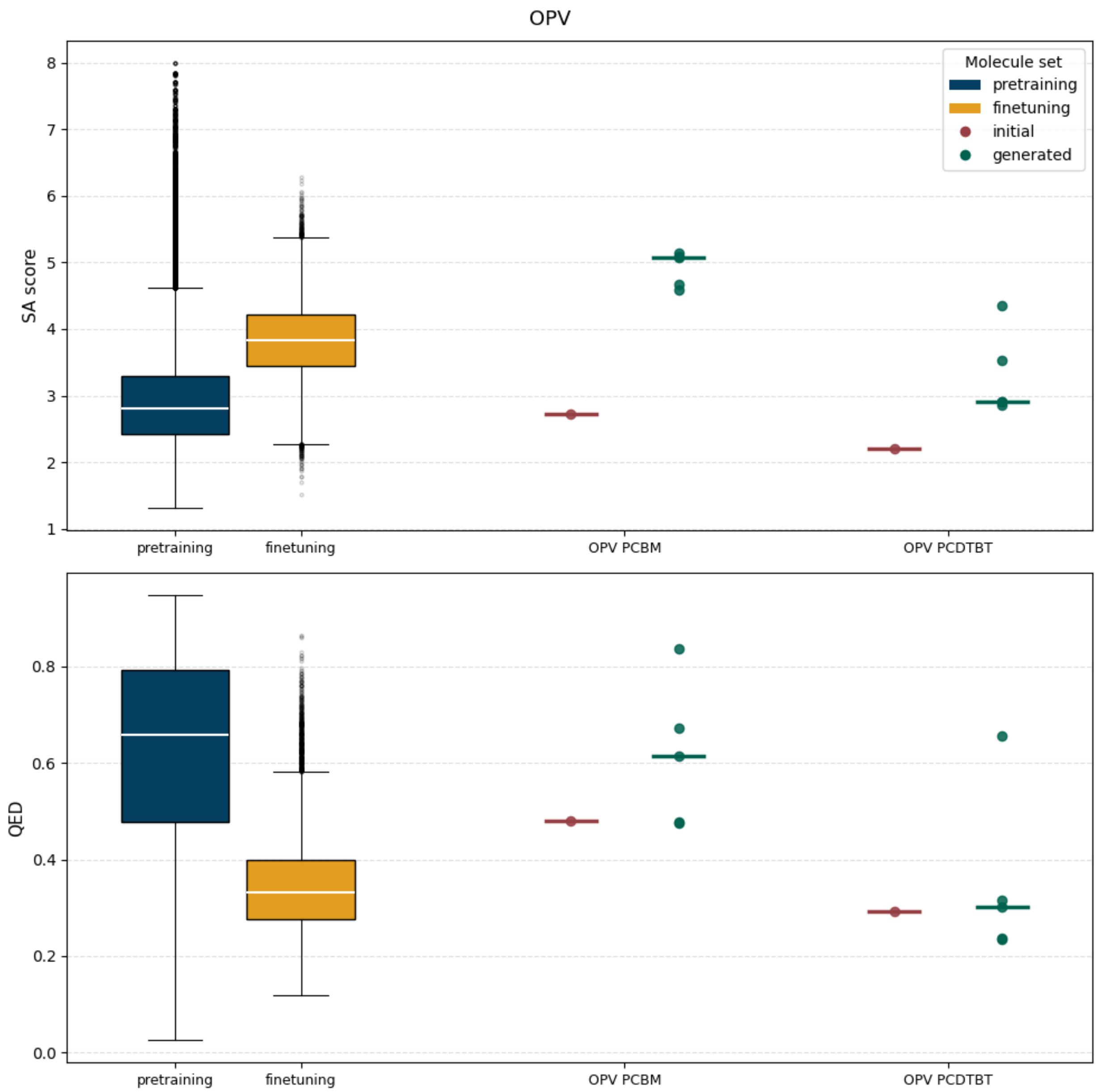

Figure S13: **SA score and QED distributions for the OPV tasks.** SA score (top) and QED (bottom) for the pretraining and finetuning datasets, initial and generated molecules for the $PCE_{PCBM} - SA_{score}$ and $PCE_{PCDTBT} - SA_{score}$ tasks.

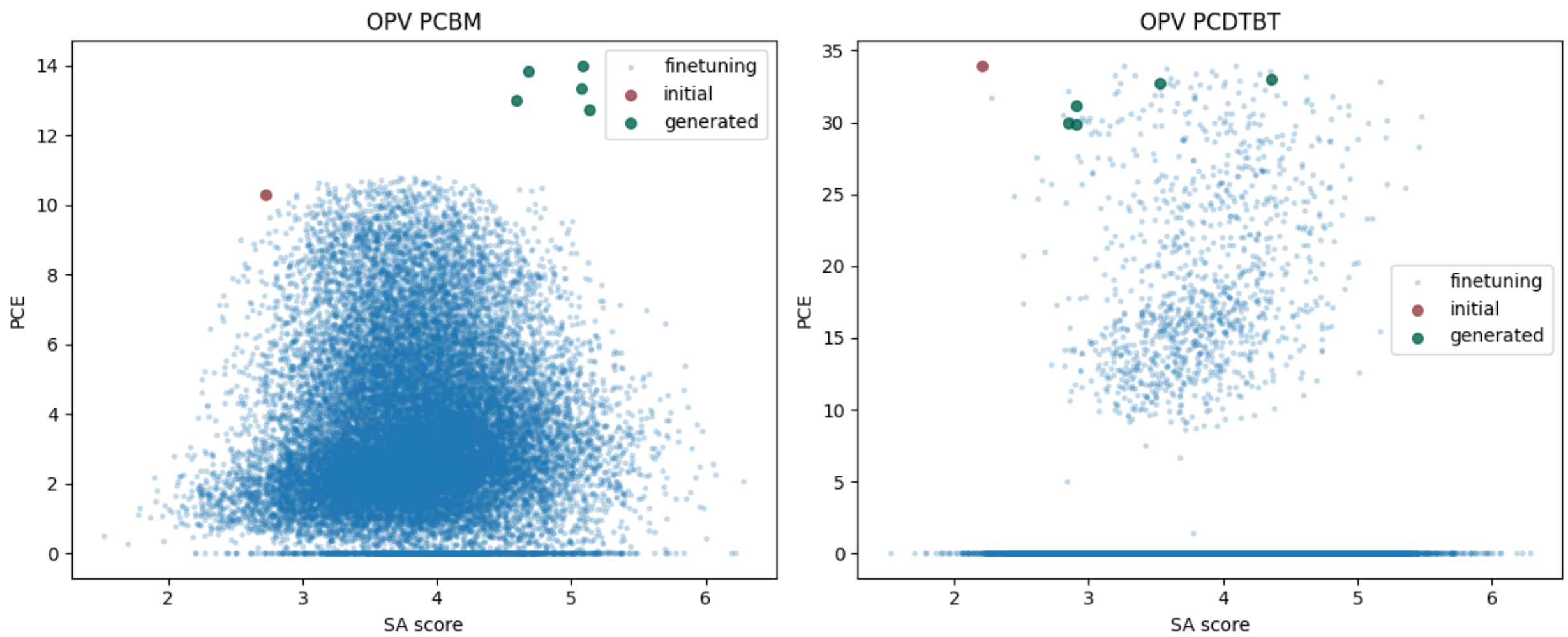

Figure S14: **PCE vs. SA score for the OPV tasks.** Power conversion efficiency (PCE) versus synthetic accessibility (SA score) with respect to PCBM (left) and PCDTBT (right) for molecules in the fine-tuning task dataset (blue), initial molecules (red) and best molecules generated by chembed (green)

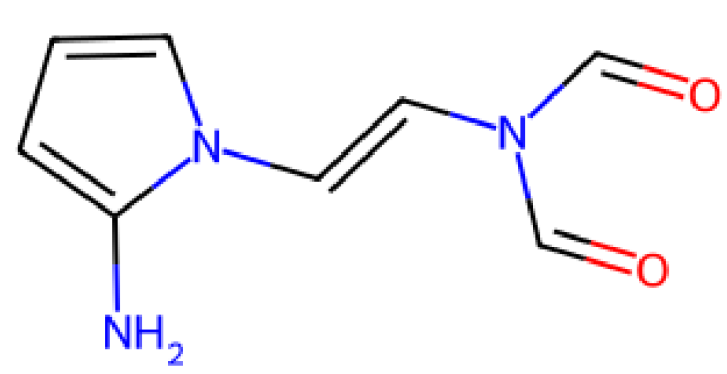

(a) Fitness: 0.0045
SA_{score}: 4.18
Tanimoto: 0.27

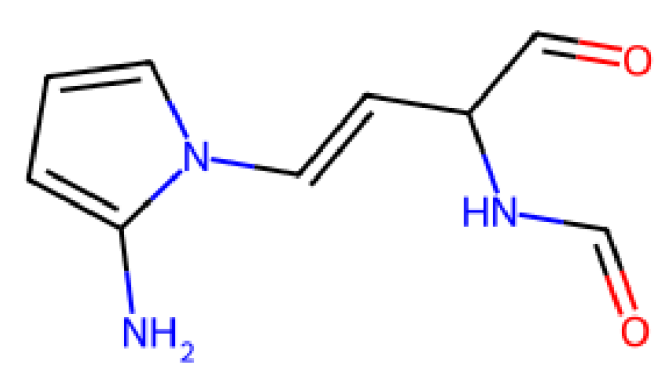

(b) Fitness: 0.0034
SA_{score}: 4.50
Tanimoto: 0.25

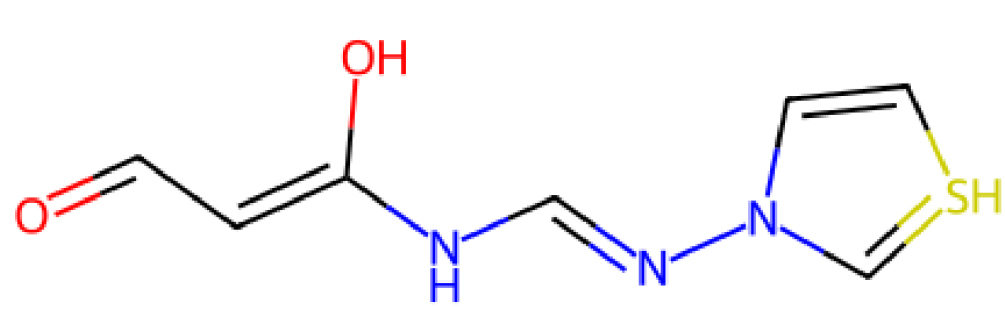

(c) Fitness: 7×10^{-15}
SA_{score}: 5.62
Tanimoto: 0.09

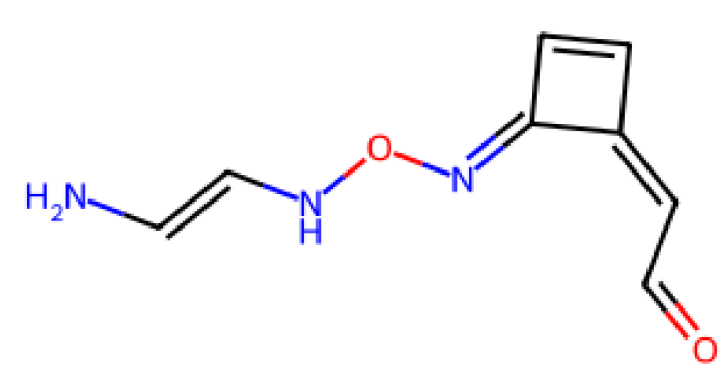

(d) Fitness: 0.0015
SA_{score}: 4.78
Tanimoto: 0.09

Figure S15: **Generated molecules for the singlet-triplet task.**Molecules generated by our model for the 5 independent runs on the singlet-triplet minimization task. Molecule (a) was proposed twice.

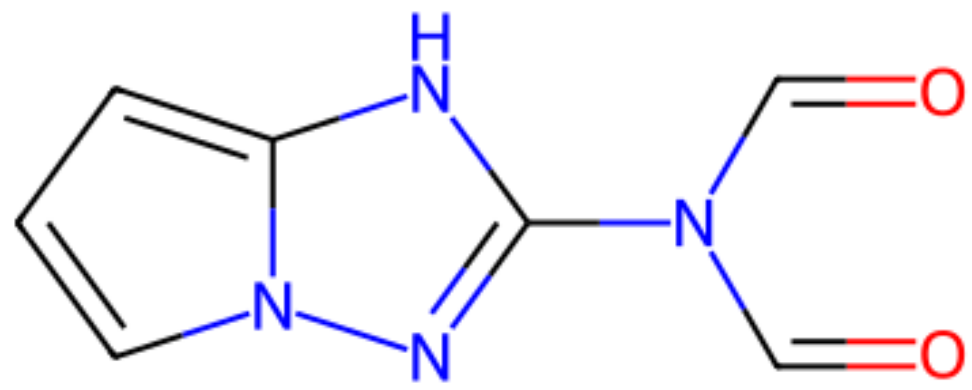

Figure S16: **Best dataset molecules for the singlet-triplet task.** Molecule in the dataset with the highest fitness score for the singlet-triplet task, with fitness score 0.020 and SA_{score} 4.03

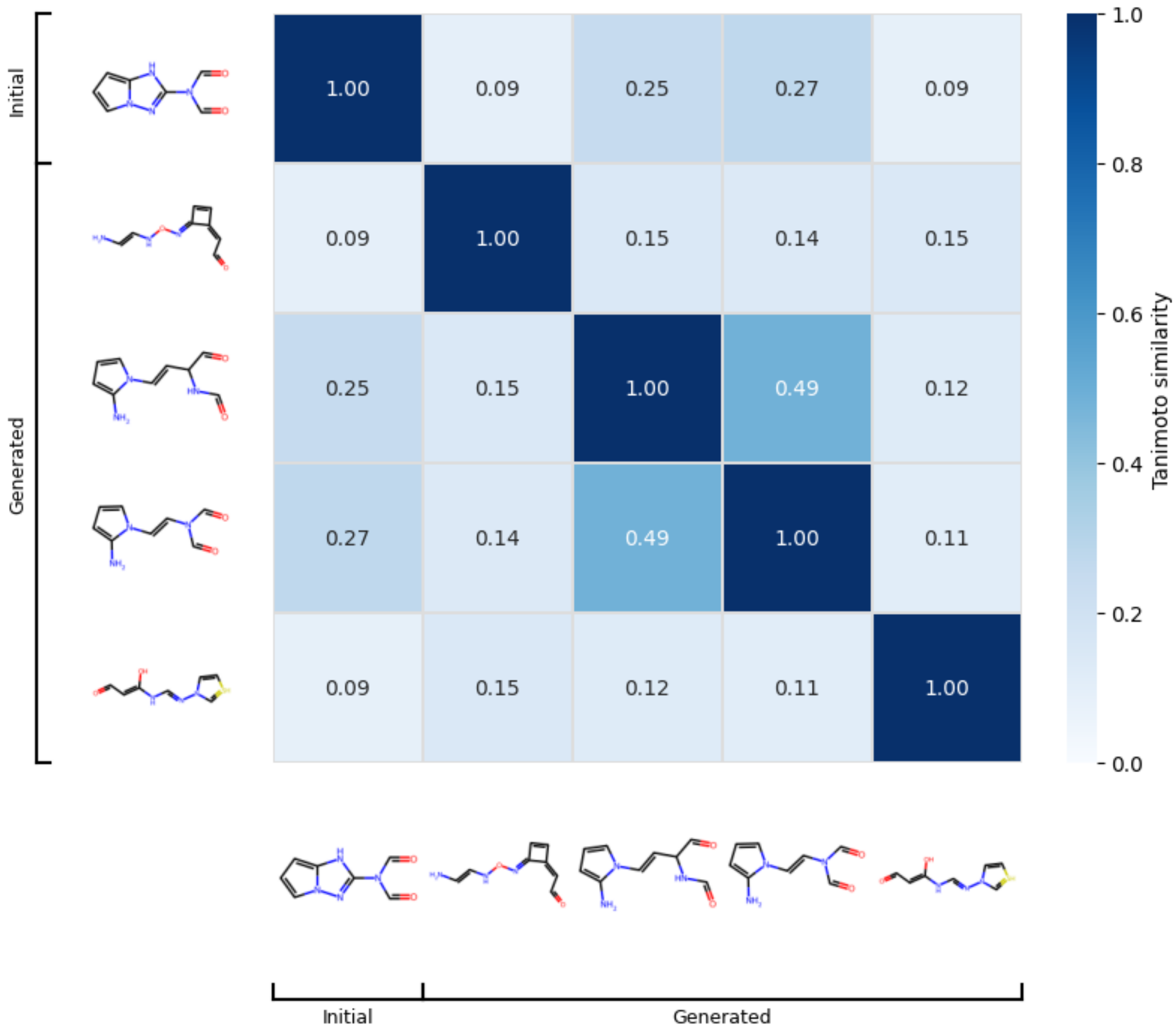

Figure S17: **Tanimoto similarity matrix for the singlet-triplet minimization task.** Matrix of pairwise Tanimoto similarities between initial and generated molecules for the singlet-triplet task.

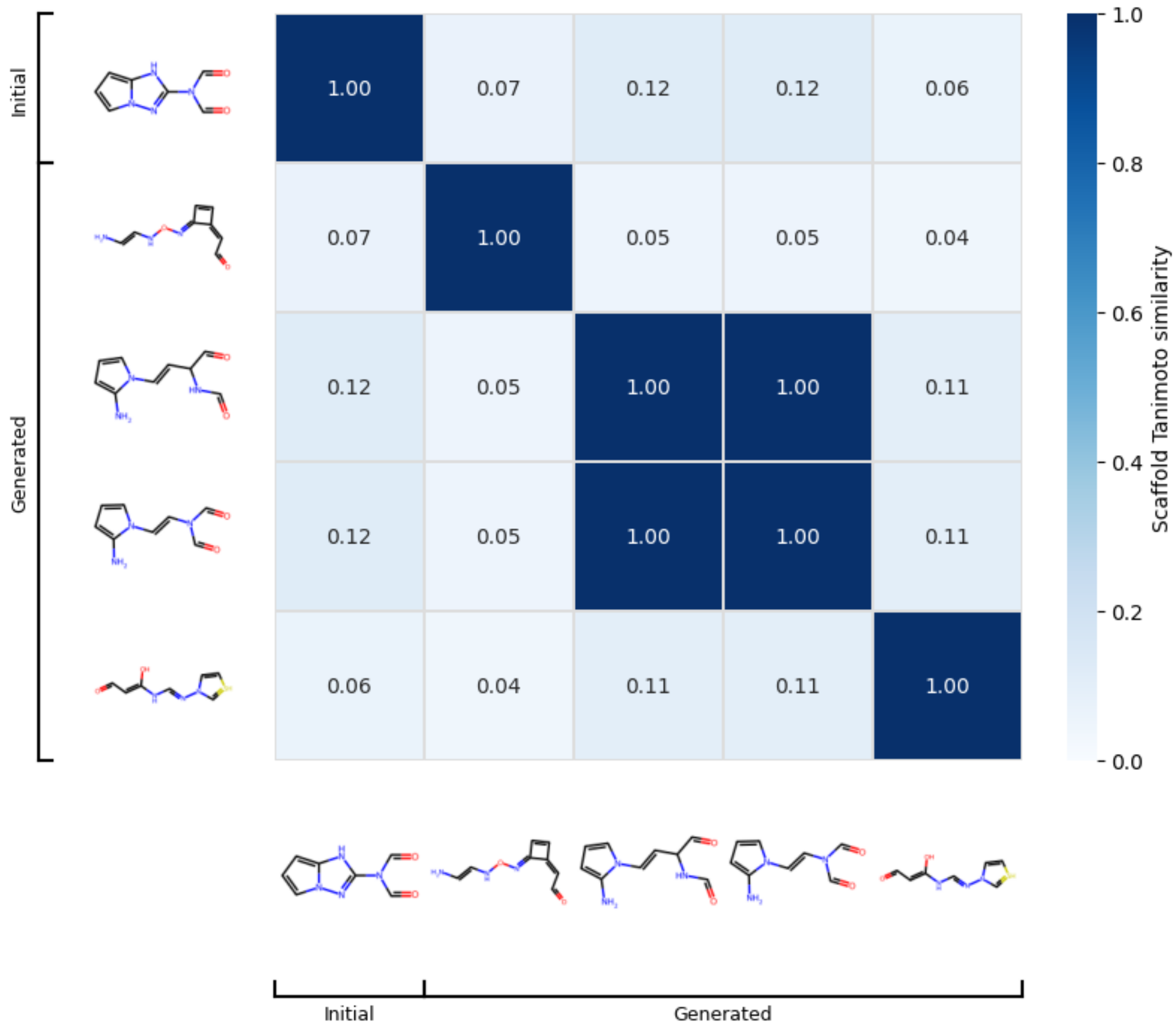

Figure S18: **Scaffold similarity matrix for the singlet-triplet minimization task.** Matrix of pairwise scaffold similarities between initial and generated molecules for the singlet-triplet task.

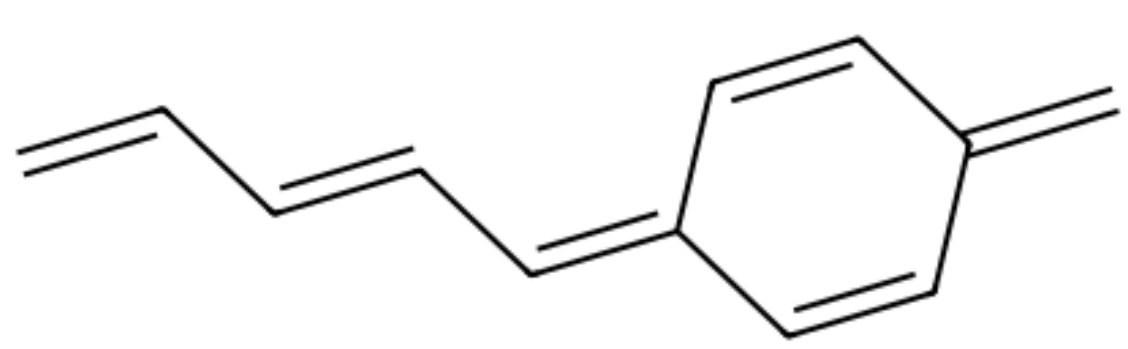

Figure S19: **Best dataset molecules for the oscillator strength task.** Molecule in the dataset with the highest fitness score for the oscillator strength maximization task, with fitness score 2.97 and SA_{score} 3.79

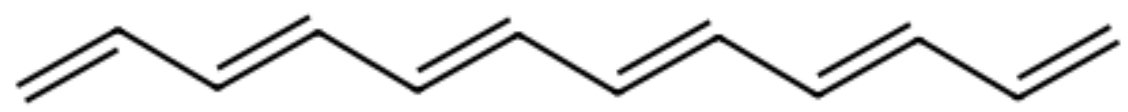

Figure S20: **Generated molecules for the oscillator strength task.** Molecule generated by our model in 3 out of 5 runs of the oscillator strength maximization task, with fitness 3.68, SA_{score} 3.41 and a Tanimoto similarity of 0.33 with the initial molecule

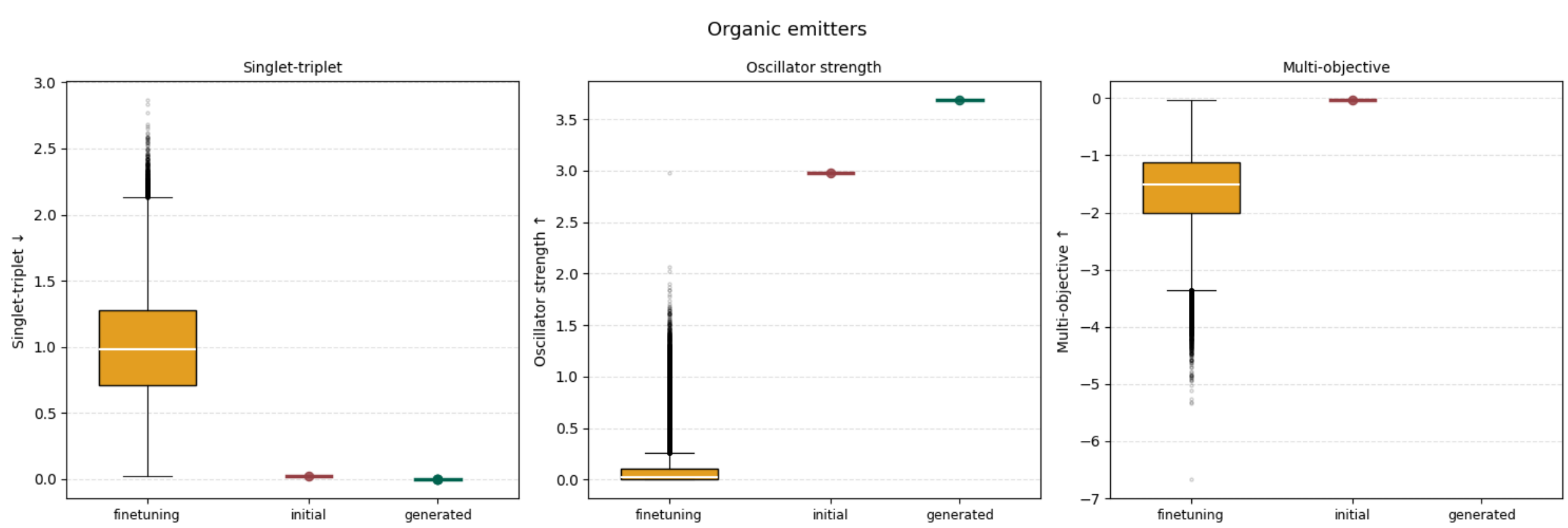

Figure S21: **Fitness distributions for the organic emitter tasks.** Fitness distributions for the finetuning dataset, initial and generated molecules for the singlet-triplet, oscillator strength and multi-objective tasks.

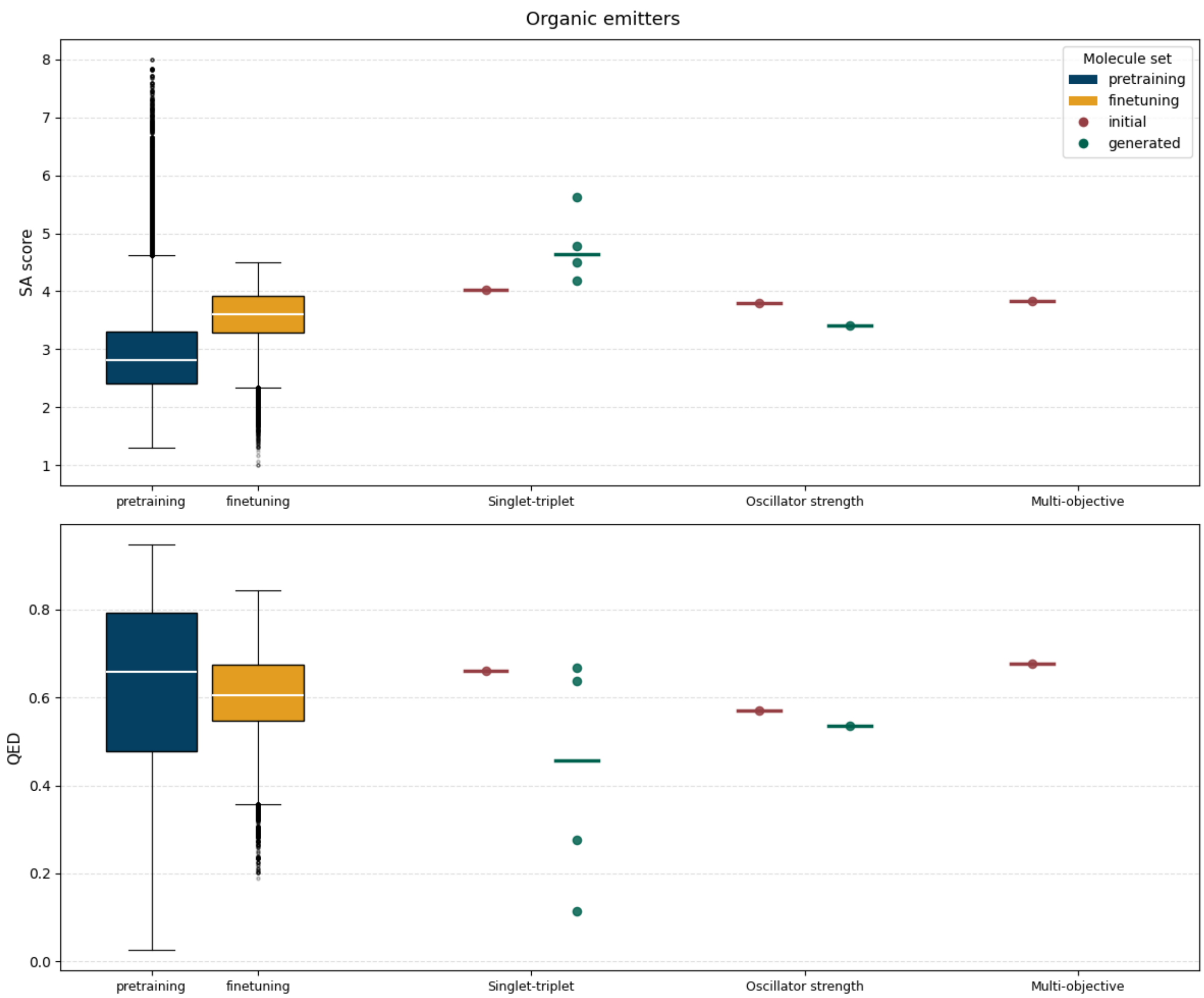

Figure S22: **SA score and QED distributions for the organic emitter tasks.** SA score (top) and QED (bottom) for the pretraining and finetuning datasets, initial and generated molecules for the singlet-triplet, oscillator strength and multi-objective tasks.

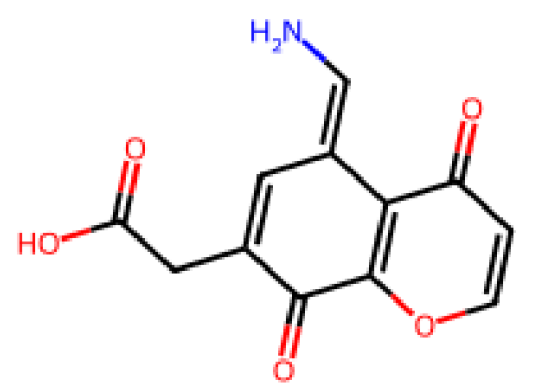

(a) Fitness: -10.7
SA_{score}: 3.45
Tanimoto: 0.26

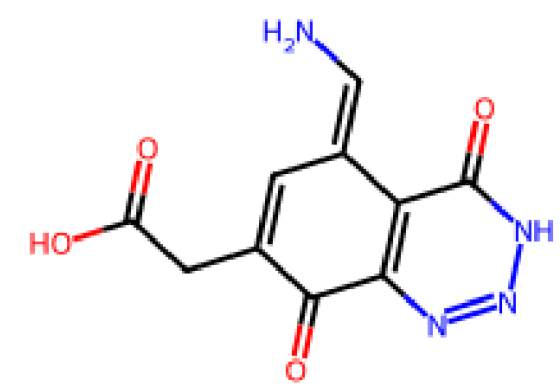

(b) Fitness: -10.7
SA_{score}: 3.75
Tanimoto: 0.26

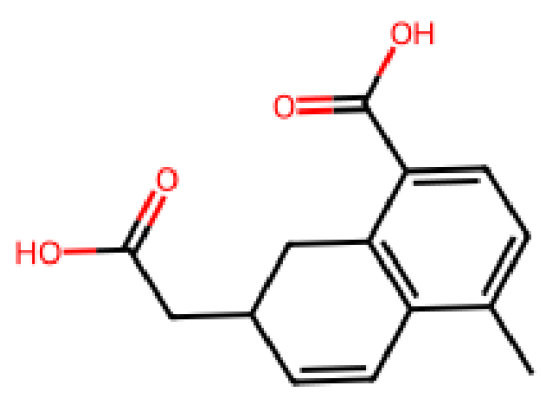

(c) Fitness: -10.7
SA_{score}: 3.33
Tanimoto: 0.26

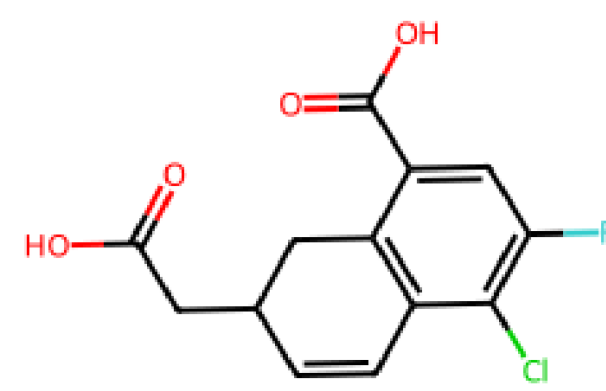

(d) Fitness: -10.7
SA_{score}: 3.54
Tanimoto: 0.23

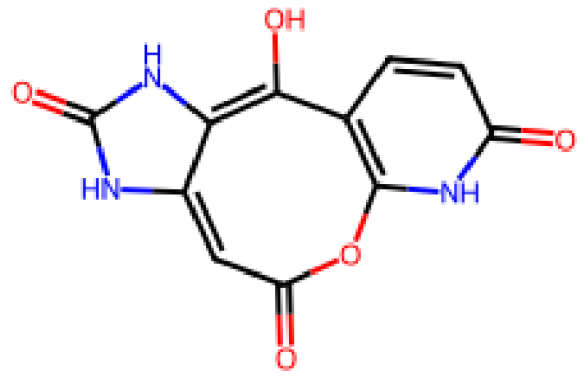

(e) Fitness: -11.2
SA_{score}: 3.78
Tanimoto: 0.25

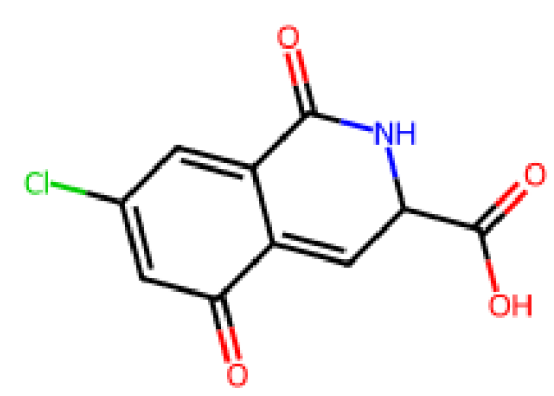

(f) Fitness: -11.1
SA_{score}: 1.90
Tanimoto: 0.18

Figure S23: **Generated molecules for the 1SYH task.** Molecules generated by our model in the 1SYH docking task

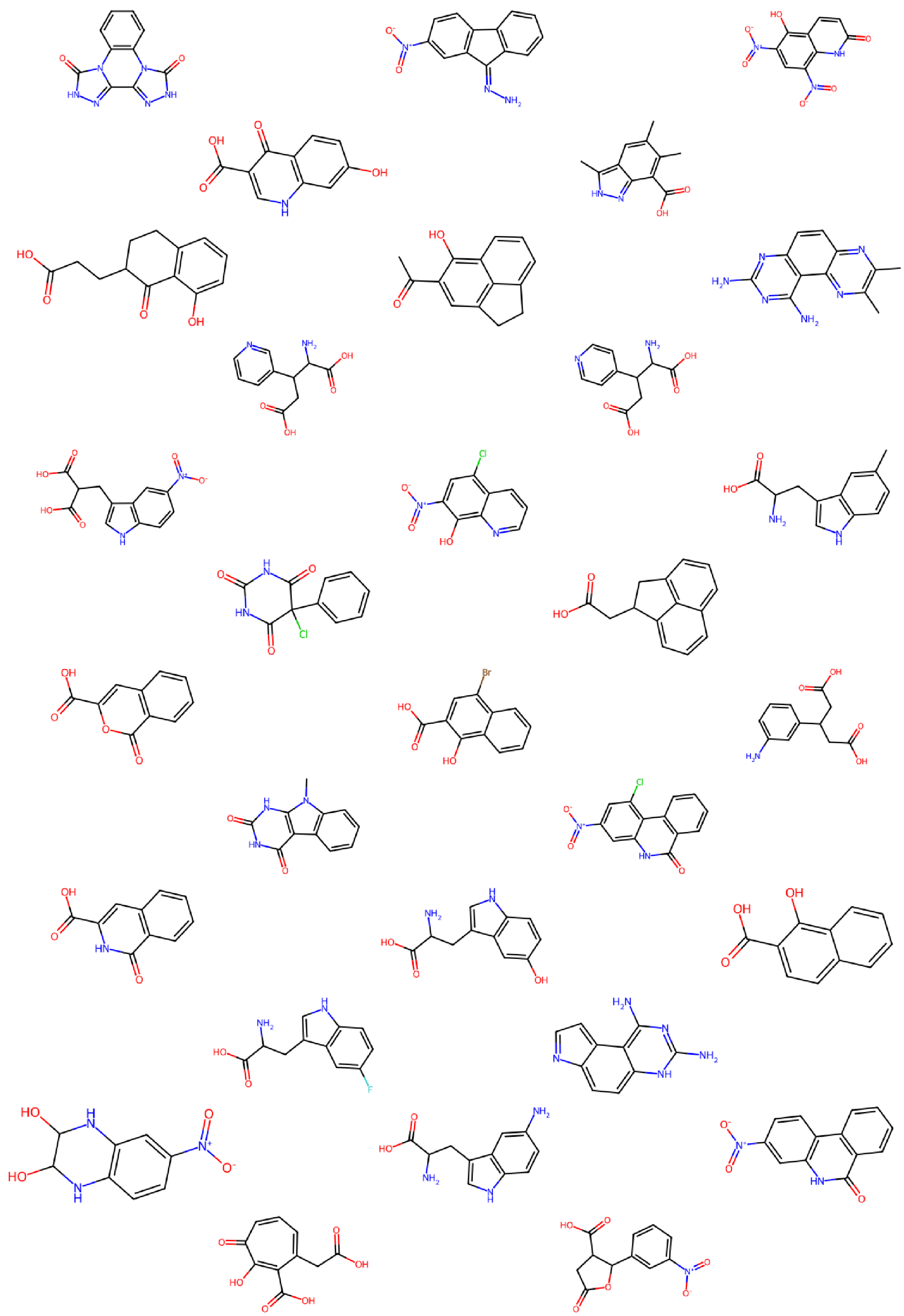

Figure S24: **Best dataset molecules for the 1SYH task.** All molecules with the best affinity score with 1SYH (−9.9) in the initial dataset

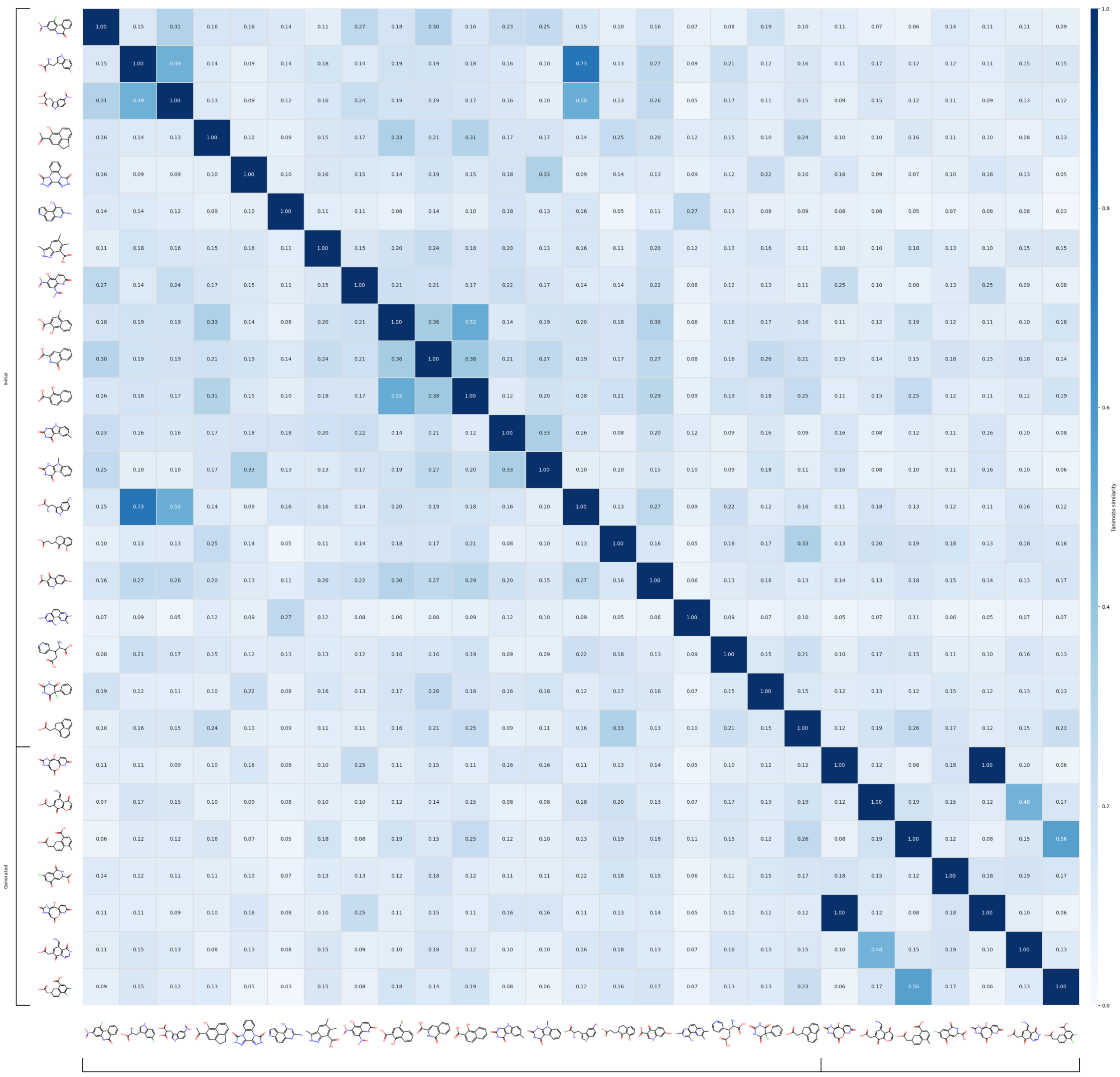

Figure S25: **Tanimoto similarity matrix for the 1SYH task.** Matrix of pairwise Tanimoto similarities between initial and generated molecules for the 1SYH task.

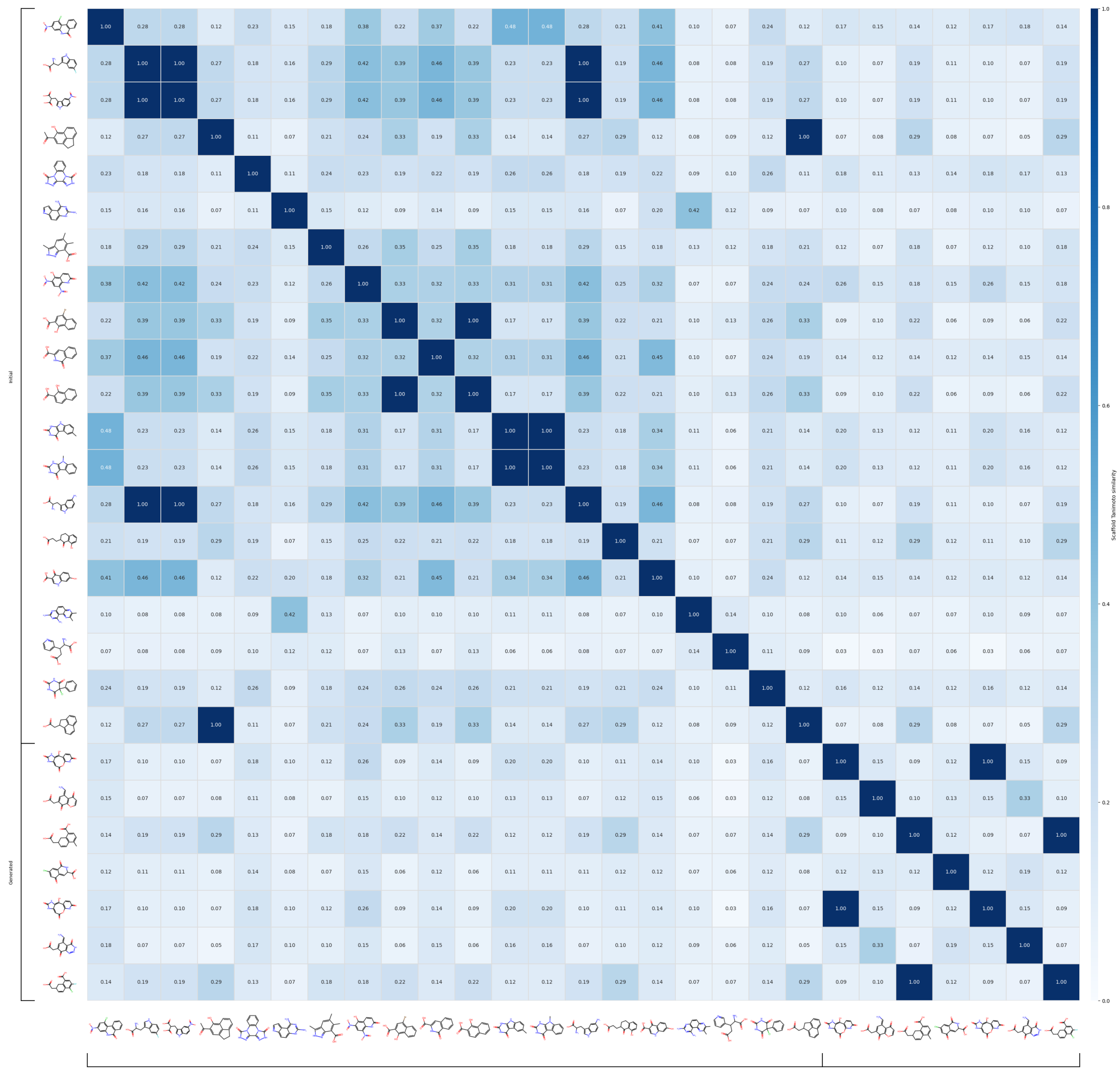

Figure S26: **Scaffold similarity matrix for the 1SYH task.** Matrix of pairwise scaffold similarities between initial and generated molecules for the 1SYH task.

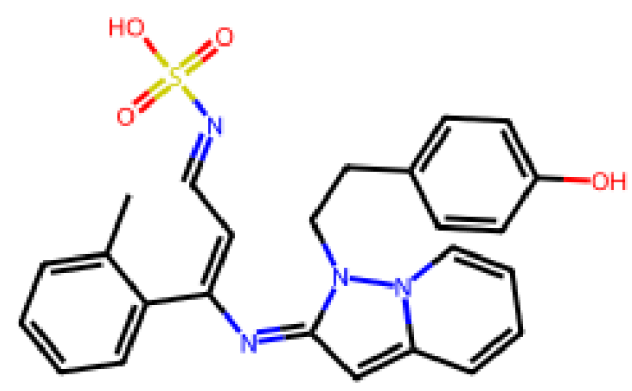

(a) Fitness: −7.5
SA_{score}: 3.57
Tanimoto: 0.14

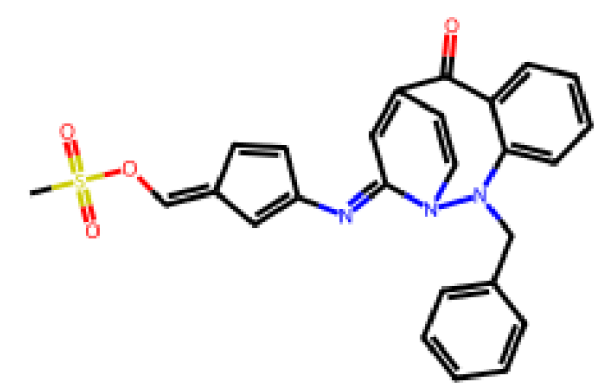

(b) Fitness: −7.5
SA_{score}: 4.29
Tanimoto: 0.21

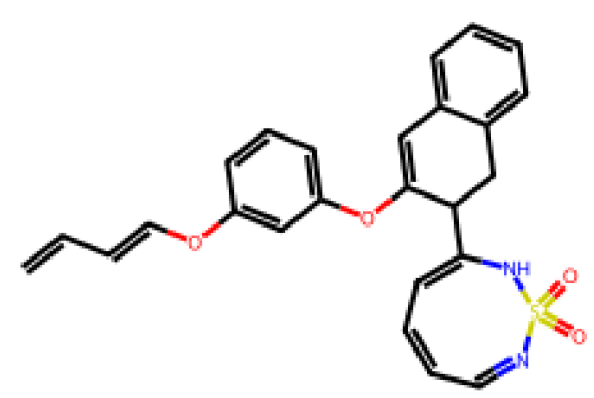

(c) Fitness: −7.8
SA_{score}: 4.28
Tanimoto: 0.09

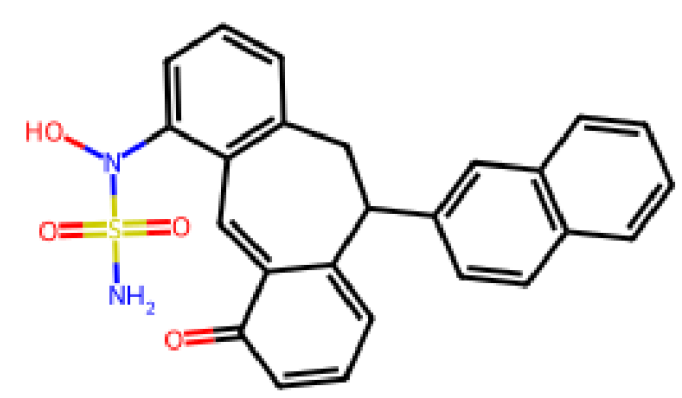

(d) Fitness: −8.6
SA_{score}: 3.68
Tanimoto: 0.12

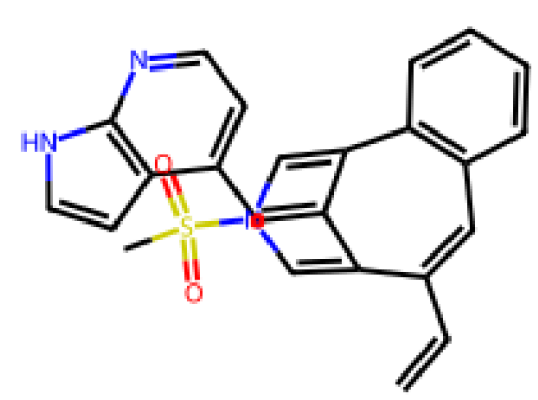

(e) Fitness: −7.7
SA_{score}: 2.30
Tanimoto: 0.12

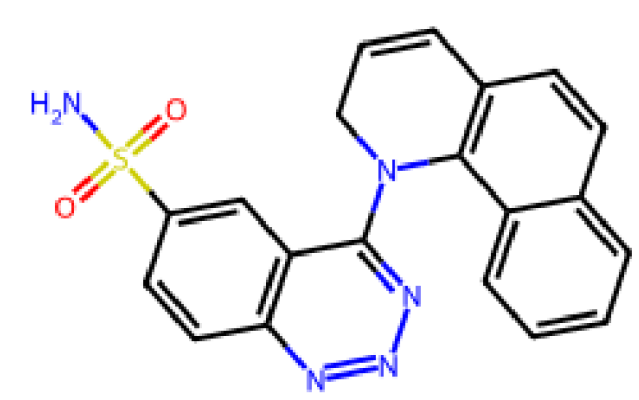

(f) Fitness: −8.3
SA_{score}: 2.89
Tanimoto: 0.27

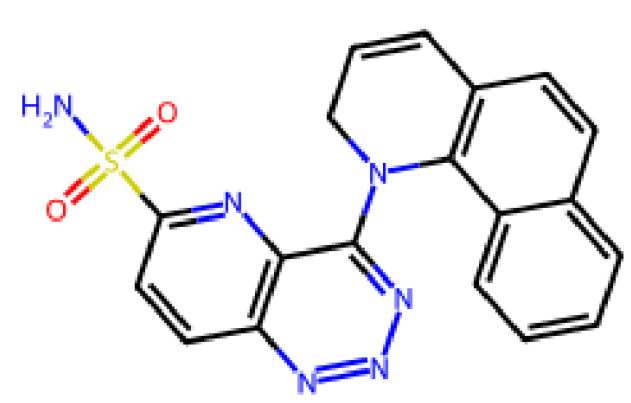

(g) Fitness: −8.3
SA_{score}: 2.30
Tanimoto: 0.20

Figure S27: **Generated molecules for the 6Y2F task**. Molecules generated by our model in the 6Y2F docking task

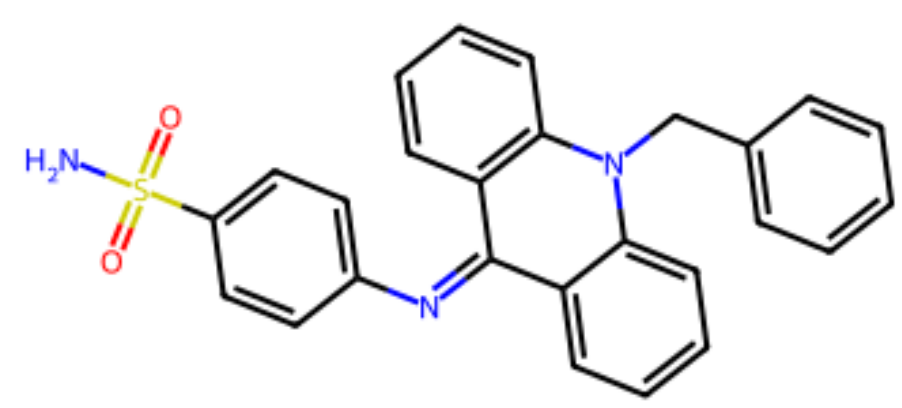

Figure S28: **Best molecule in the dataset for the 6Y2F task.** Molecule in the dataset with the best affinity with 6Y2F (Fitness: -8.2, SA_{score}: 2.30)

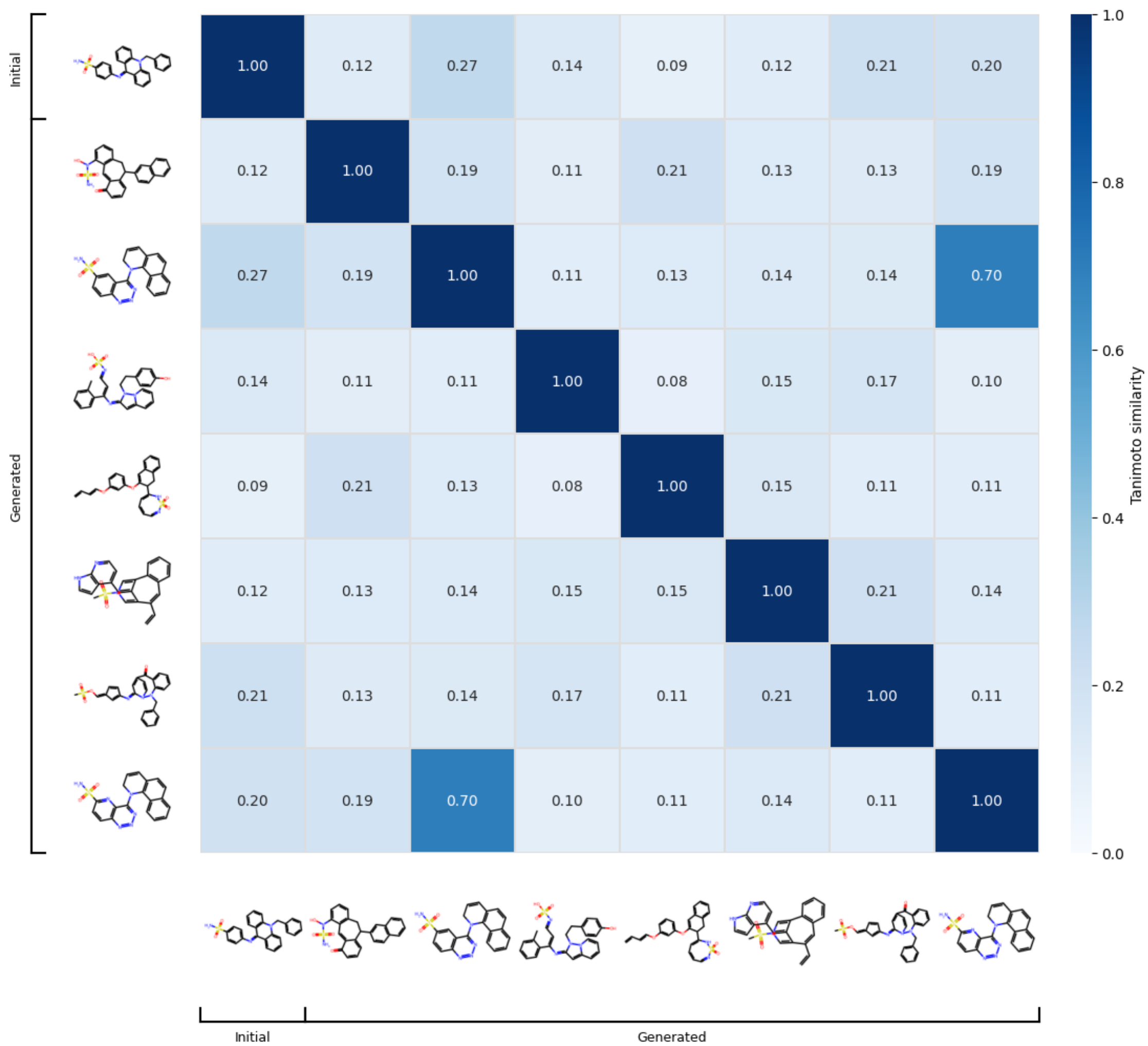

Figure S29: **Tanimoto similarity matrix for the 6Y2F task.** Matrix of pairwise Tanimoto similarities between initial and generated molecules for the 6Y2F task.

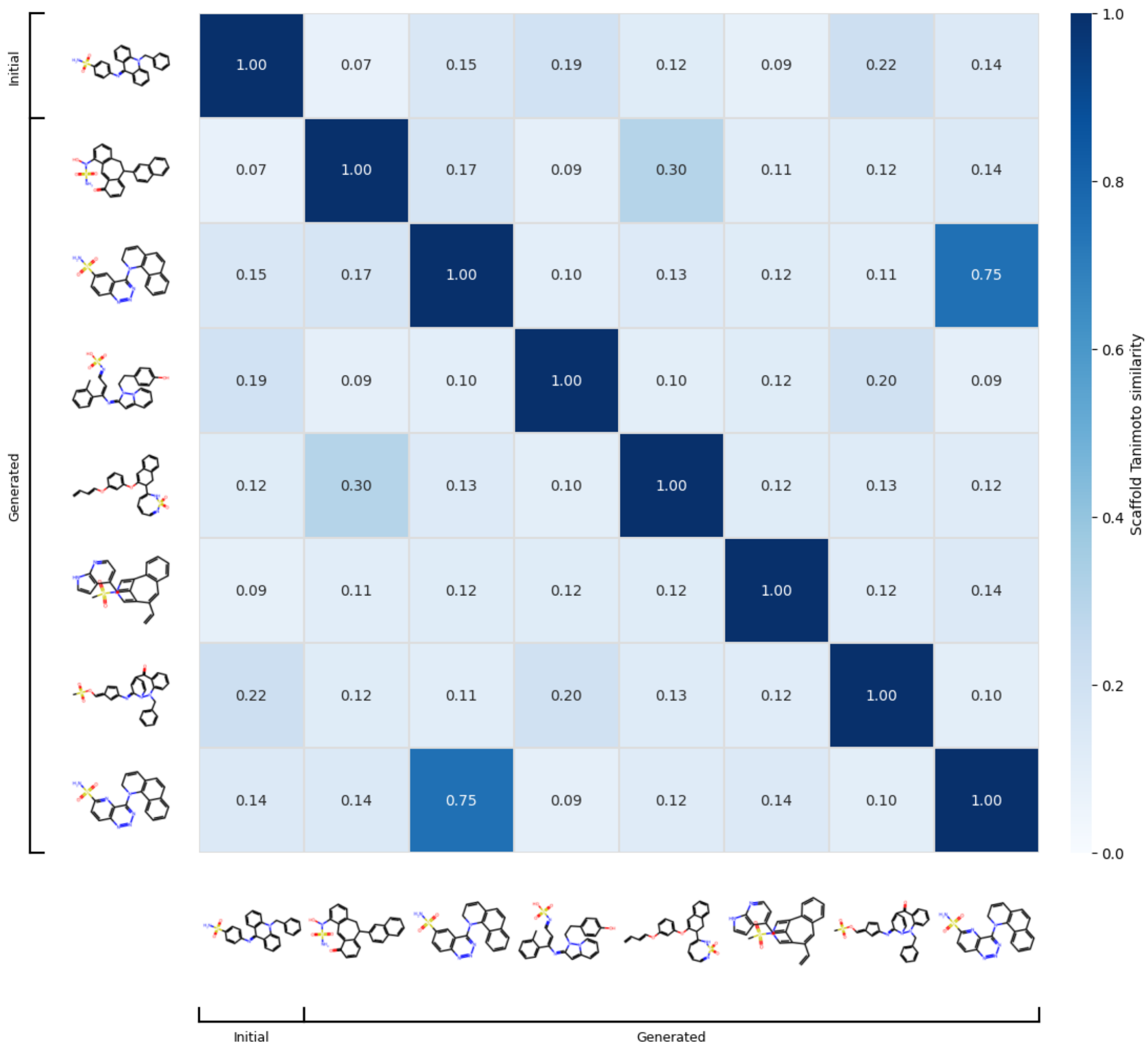

Figure S30: **Scaffold similarity matrix for the 6Y2F task.** Matrix of pairwise scaffold similarities between initial and generated molecules for the 6Y2F task.

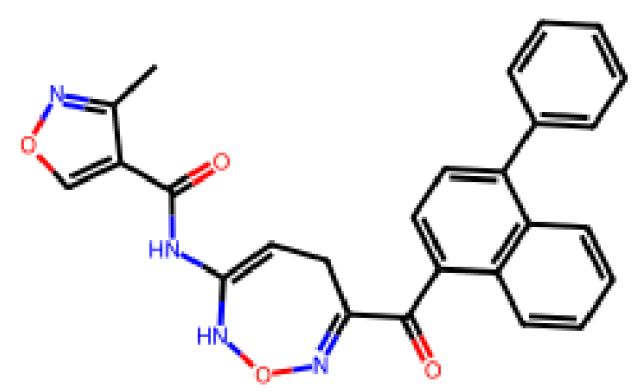

(a) Fitness: -13.6
SA_{score}: 3.16
Tanimoto: 0.29

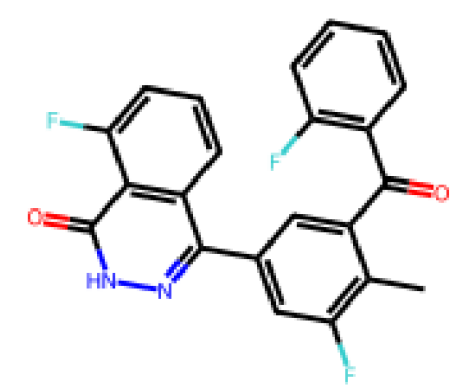

(b) Fitness: -14.6
SA_{score}: 2.53
Tanimoto: 0.34

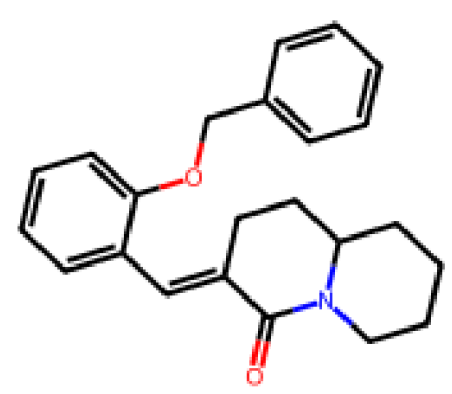

(c) Fitness: -12.3
SA_{score}: 2.59
Tanimoto: 0.36

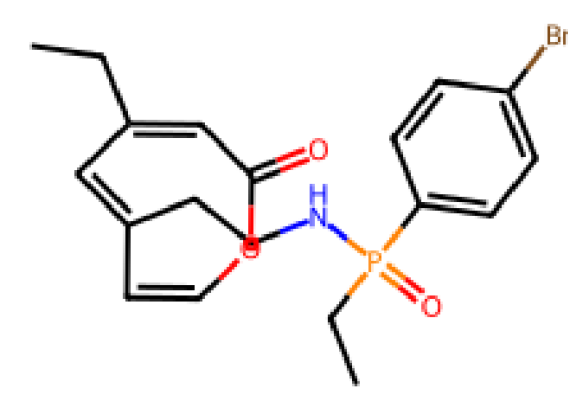

(d) Fitness: -11.9
SA_{score}: 4.30
Tanimoto: 0.20

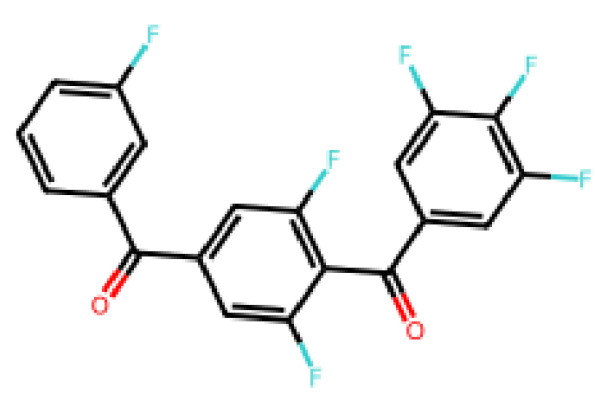

(e) Fitness: -13.3
SA_{score}: 2.35
Tanimoto: 0.37

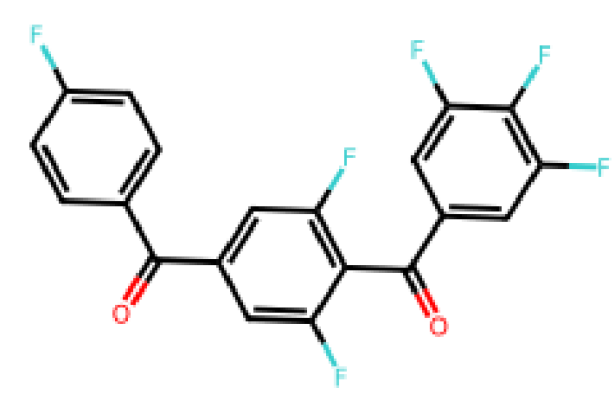

(f) Fitness: -13.3
SA_{score}: 2.27
Tanimoto: 0.39

Figure S31: **Generated molecules for the 4LDE task.** Molecules generated by our model in the 4LDE docking task

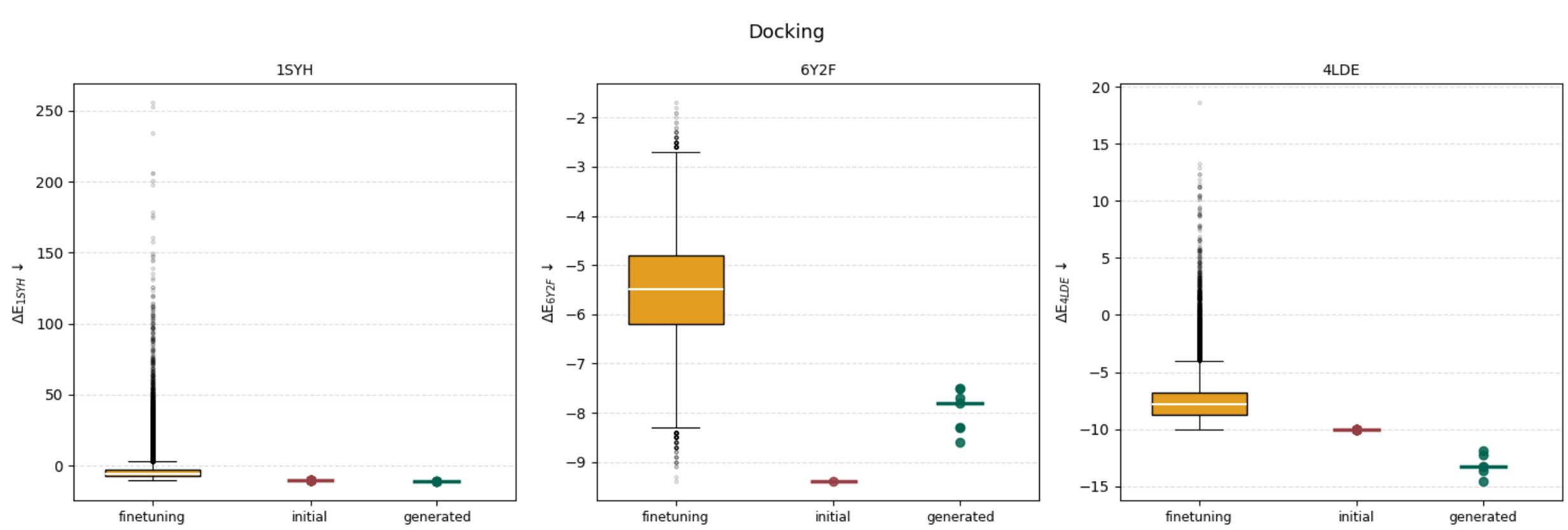

Figure S32: **Fitness distributions for the docking tasks.** Fitness distributions for the finetuning dataset, initial and generated molecules for the 1SYH, 6Y2F and 4LDE tasks.

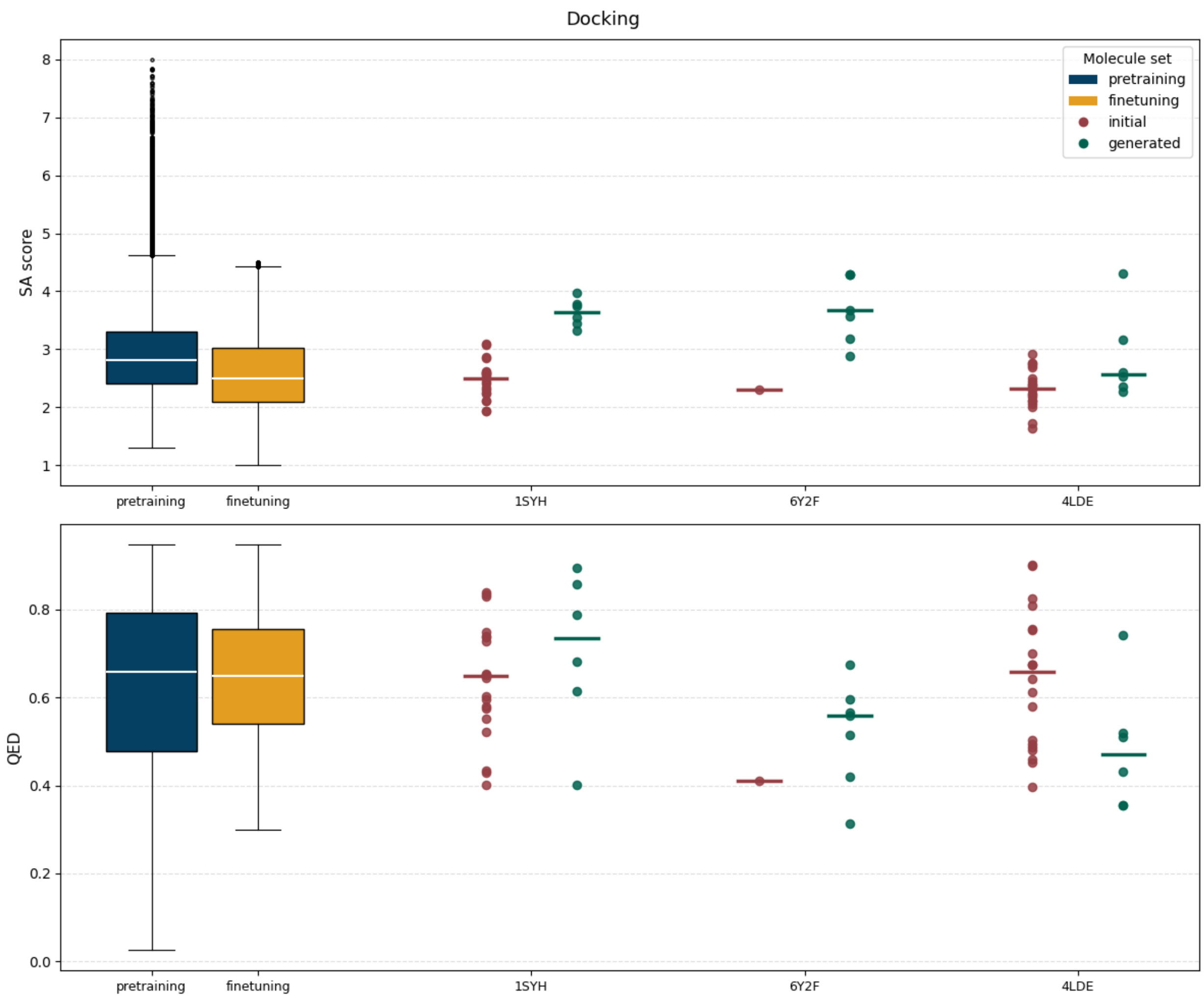

Figure S33: **SA score and QED distributions for the docking tasks.** SA score (top) and QED (bottom) for the pretraining and finetuning datasets, initial and generated molecules for the 1SYH, 6Y2F and 4LDE tasks.

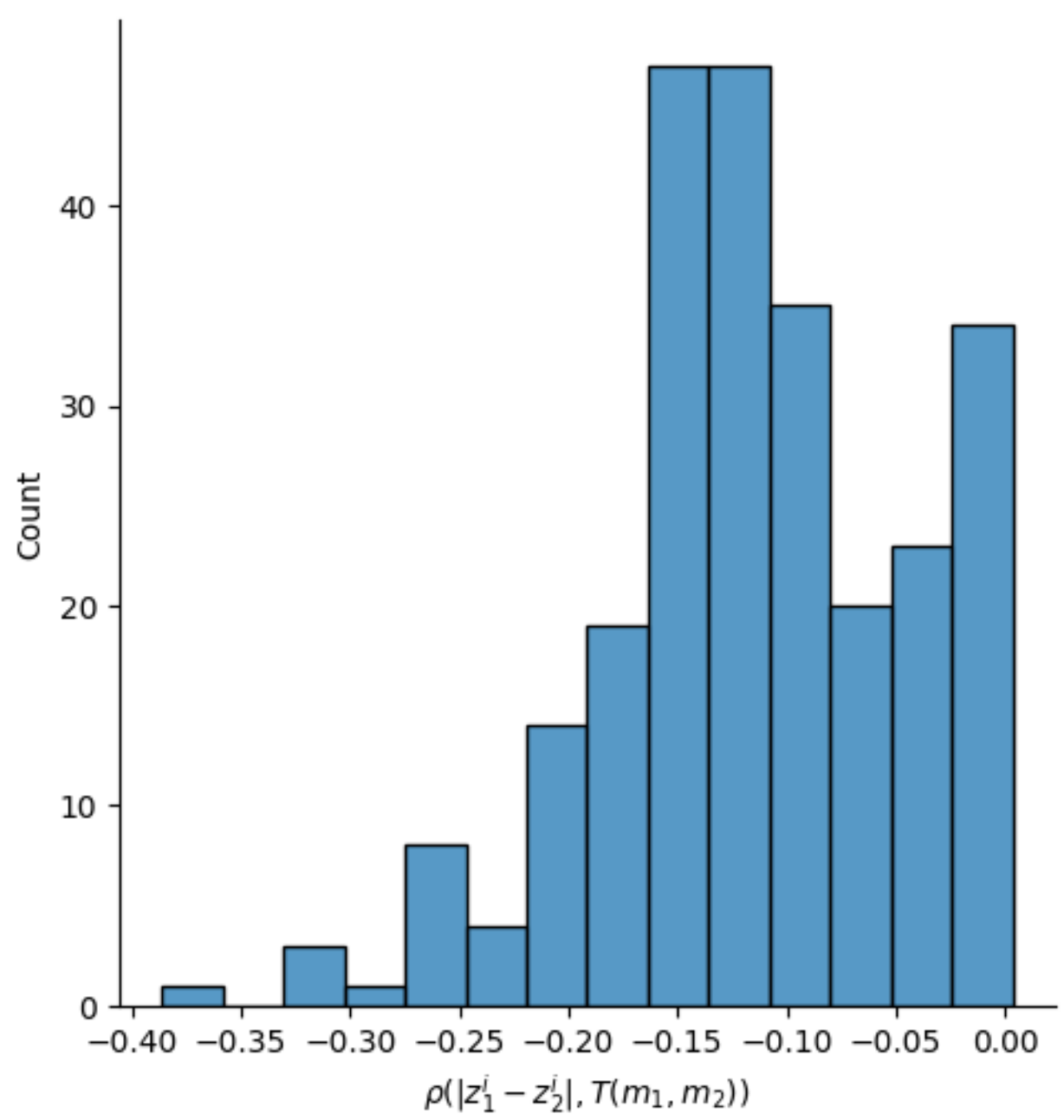

Figure S34: **Pearson correlation between absolute latent differences and Tanimoto similarity across latent dimensions.** Distribution of Pearson correlations between absolute latent vector differences along each dimension $i \in \{1, \cdots, 256\}$ $|z_1^i - z_2^i|$ and Tanimoto similarities between initial molecules $T(m_1, m_2)$ for 1 000 000 random molecule pairs sampled from the test set

References

[1] Guy W Bemis and Mark A Murcko. The properties of known drugs. 1. molecular frameworks. *Journal of medicinal chemistry*, 39(15):2887–2893, 1996.

[2] Jarkko Venna and Samuel Kaski. Neighborhood preservation in nonlinear projection methods: An experimental study. In *Proceedings of the International Conference on Artificial Neural Networks*, pages 485–491, Berlin, Heidelberg, 2001. Springer-Verlag.
